# Development and evaluation of a practical teaching model for medical laboratory interns based on the “quality-safety-patient-centered” triple helix framework

**DOI:** 10.3389/fmed.2026.1936253

**Published:** 2026-10-14

**Authors:** Qin Wang, Qiang Zhou, Xiu Wang, Wei Tang, Naifang Ye, Aihua Wang, Hao Zhang, Jie Yao

**Affiliations:** Department of Clinical Laboratory, The Second Affiliated Hospital of Anhui Medical University, Hefei, China

**Keywords:** clinical internship program, competency-based education, laboratory medicine, patient-centered care, quality improvement, teaching reform

## Abstract

**Background:**

Balancing competency-based education, quality improvement, and patient-centered care remains a challenge in laboratory medicine internships. This study developed a “quality–safety–patient-centered” triple helix framework and evaluated its association with educational outcomes in an exploratory quasi-experimental setting.

**Methods:**

This study employed a two-phase, quasi-experimental, non-randomized design with a historical control group. The control group (*n* = 30, grade 2022) received conventional teaching, while the experimental group (*n* = 32, grade 2023) underwent the Triple Helix–based model. Both completed a 48-week internship. Outcomes included the Triple Helix Integrated Literacy Scale, Mini-Clinical Evaluation Exercise (Mini-CEX), theoretical and practical examinations, process quality and safety indicators, and satisfaction ratings.

**Results:**

The experimental group showed higher scores than the control group on the Triple Helix Integrated Literacy Scale total score (106.25 ± 7.96 vs. 88.70 ± 8.25; mean difference 17.55, 95% CI: 13.42–21.66; *P* < 0.001), Mini-CEX comprehensive score (7.63 ± 0.53 vs. 6.47 ± 0.62; mean difference 1.16, 95% CI: 0.88–1.44; *P* < 0.001), theoretical examination score (87.91 ± 4.98 vs. 82.47 ± 5.86; mean difference 5.44, 95% CI: 2.67–8.21; *P* < 0.001), and practical skills (90.28 ± 4.87 vs. 84.13 ± 6.02; mean difference 6.15, 95% CI: 3.36–8.94; *P* < 0.001). For process indicators, the experimental group showed higher rates of quality control (QC) out-of-control management accuracy (93.8% vs. 70.0%; risk difference 23.75%, 95% CI: 5.33–42.17; *P* = 0.014), critical value notification timeliness (100% vs. 80.0%; risk difference 20.00%, 95% CI: 5.69–34.31; *P* = 0.010), and patient identification compliance rate (93.8% vs. 73.3%; risk difference 20.42%, 95% CI: 2.51–38.33; *P* = 0.040). Satisfaction ratings from clinicians, patients, and preceptors were all higher in the experimental group (all *P* < 0.001).

**Conclusion:**

In this exploratory study, the triple helix teaching model was associated with better performance across quality, safety, and patient-centered competencies. Causal inference is limited by the historical control design, yet the framework may offer a promising direction for guiding teaching reform in laboratory medicine.

## 1 Introduction

Medical laboratory science is a critical pillar of modern healthcare, as service quality directly affects clinical decision-making, patient safety, and outcomes. As laboratory medicine transitions from a purely analytical testing service toward a clinical diagnostic support platform (1), the goal has shifted from technical proficiency to comprehensive competence encompassing quality management, risk prevention, and patient-centered care (2). The clinical internship represents a pivotal stage in this transformation, during which students develop professional norms and competencies; teaching quality directly influences subsequent performance and clinical readiness (3).

Previous efforts to reform laboratory medicine internship education have largely focused on single dimensions. Quality-focused interventions have emphasized total quality management (TQM) and Six Sigma methodologies, with studies reporting improvements in interns' understanding of quality control (QC) rules and out-of-control management. Safety-focused reforms have concentrated on occupational biosafety and infection control, yet few have systematically addressed patient safety issues such as closed-loop critical value management. Patient-centered training initiatives have recently emerged, but their implementation in laboratory settings has been constrained by the limited direct patient contact inherent to the profession (4). However, to our knowledge, limited published research has systematically integrated these three dimensions into a unified training framework for laboratory interns.

The concept of a “triple helix,” as proposed by Gonzalo et al. (5) for unifying clinical, research, and education missions in academic health centers, offers a valuable analog for laboratory education. While the original model addresses institutional missions at the macro level, its core logic—synergy among distinct yet interdependent domains—is directly transferable to the micro level of internship training. Adapting this synergistic logic, we reconceptualized the triple helix as three interconnected training pillars: (1) **Quality** provides the **technical foundation**—ensuring that interns master total process quality control, from pre-analytical specimen handling to post-analytical result verification; (2) **Safety** serves as the **risk-prevention floor**—embedding patient identification, critical value reporting, and adverse event management as non-negotiable competencies; and (3) **Patient-centeredness** supplies the **value orientation**—anchoring all technical work in the ultimate goal of improving patient outcomes and experience. These three dimensions are not merely additive but **synergistic**: quality failures create safety risks, safety lapses undermine patient trust, and patient-centered awareness reinforces the importance of both quality and safety. The helix metaphor captures the iterative, upward-spiraling relationship: improvements in any one dimension potentiate gains in others.

Given these three interconnected deficiencies, a fragmented approach—addressing quality, safety, or patient-centeredness in isolation—would be unlikely to resolve the underlying educational gaps. Quality failures and safety lapses share common root causes (e.g., inadequate verification processes, communication breakdowns), and both ultimately affect patient outcomes. Conversely, patient-centered awareness can reinforce the importance of rigorous quality and safety practices by grounding them in tangible clinical consequences. This interdependence suggests that an integrated, rather than additive, training model is needed—one that systematically weaves the three dimensions into a coherent educational experience.

Despite this theoretical promise, current internship teaching remains predominantly skill-oriented (4), focusing on testing principles, instrument operation, result validation, and routine biosafety, while inadequately integrating quality management, systematic safety, and patient-centered concepts (6). Three major deficiencies are evident. First, teaching evaluations tend to overemphasize operational skills and theoretical knowledge, with insufficient assessment of total process quality control across pre-analytical, analytical, and post-analytical phases (7). Second, safety education is largely confined to occupational exposure and laboratory protection, failing to adequately address core patient safety issues such as patient identification, closed-loop critical value management, and laboratory information transmission (8). Third, owing to the nature of the profession, students interact more with specimens and data than with patients, which may foster a cognitive bias toward “specimen-centered” rather than “patient-centered” practice, thereby hindering the formation of a patient-centered professional value system.

In recent years, promoting a patient-centered transformation in laboratory medicine has become a growing consensus. As advocated by ISO 15189, medical laboratories must build quality and competence management systems that closely align with patient needs (9, 10). International standards and hospital accreditation criteria emphasize that laboratory value extends beyond analytical accuracy to total process quality control, clinical decision support, and patient experience and safety (11). Given this trend, the traditional skill-oriented internship model is clearly inadequate for meeting current demands in laboratory professional development. Therefore, this study introduces a “quality–safety–patient-centered” triple helix framework. Drawing on Gonzalo et al.'s triple helix concept (5), we systematically integrated quality management, safety governance, and patient-centered services throughout the internship. A quasi-experimental design was employed to evaluate the association of this framework with educational outcomes and to provide preliminary empirical data for clinical teaching reform in laboratory medicine.

## 2 Subjects and methods

### 2.1 Subjects

This study enrolled medical laboratory undergraduate interns from the grades of 2022 and 2023 at the Second Affiliated Hospital of Anhui Medical University. The control group comprised 30 interns from the grade of 2022, and the experimental group comprised 32 interns from the grade of 2023. Inclusion criteria were: (1) completion of the full 48-week clinical internship; (2) complete assessment data; and (3) provision of informed consent with voluntary participation. Exclusion criteria were withdrawal from the internship or missing key research data. No significant differences were observed between groups in measured baseline characteristics, including age, sex, pre-internship theoretical and practical scores (all *P* > 0.05), suggesting no obvious imbalance (Table 3).

Given the quasi-experimental, non-randomized design with historical controls based on consecutive internship cohorts, a conventional *a priori* sample size calculation was not performed. The sample comprised all eligible interns from the 2022 (*n* = 30) and 2023 (*n* = 32) cohorts, representing the full population of interns during the study period. Given the exploratory nature of this study and the fixed cohort sizes, formal power calculation was not performed; the emphasis is placed on effect estimates and confidence intervals rather than *P*-values alone.

### 2.2 Study design

This study employed a quasi-experimental design with non-contemporaneous historical controls. The control group received the conventional teaching model traditionally used in the department, while the experimental group received the modified teaching model based on the “quality-safety-patient-centered” triple helix framework. Both groups had identical rotation schedules across subspecialties (including clinical hematology and body fluid analysis, clinical chemistry, immunology, microbiology, molecular diagnostics, and outpatient/emergency laboratory services) and the same internship duration.

To contextualize the historical control design, we assessed the stability of key teaching and clinical environment factors between 2022 and 2023. The rotation schedules, subspecialty placement durations, and examination formats (both theoretical and practical) remained unchanged between the two years. The same 40 preceptors participated in teaching across both cohorts; no major faculty turnover occurred during the study period. Departmental standard operating procedures, quality control protocols, and critical value reporting policies remained consistent, as the department maintained ISO15189 accreditation continuously throughout both years.

However, we acknowledge that certain unmeasured factors—including subtle annual variations in hospital-wide quality campaigns, preceptor teaching experience accrual, cohort-specific learning motivation, and external clinical workload fluctuations—may have differed between the two periods and could not be fully controlled. Nonetheless, temporal confounding cannot be entirely excluded. Given these constraints, the historical control design was chosen for feasibility, and the findings are intended to inform future prospective validation rather than to establish definitive causal effects.

### 2.3 Teaching methods

#### 2.3.1 Control group

The conventional teaching model was followed. Preceptors in each subspecialty delivered instruction according to the established rotation plan, covering testing principles, instrument operation procedures, basic internal quality control rules, and biosafety protocols. Teaching was delivered through lectures, instrument demonstrations, and supervised practical sessions, following standard rotation syllabi. Interns received routine preceptor feedback on their performance during each rotation, but no systematic case-based discussions, scenario simulations, or structured quality–safety–patient-centered content were integrated into their teaching. This approach served as the control condition against which the triple helix intervention was compared.

#### 2.3.2 Experimental group

The “quality–safety–patient-centered” triple helix framework was embedded into routine daily clinical instruction. Throughout each 8-week rotation, preceptors integrated quality, safety, and patient-centered content into existing teaching activities—including result verification sessions, QC review meetings, specimen reception walk-throughs, and phlebotomy supervision. Each rotation included approximately 8–10 embedded teaching encounters across the three dimensions, each lasting 15–30 min as part of routine workflow. No additional teaching time was allocated; rather, existing instructional contacts were repurposed to include Triple Helix content, ensuring equal total instructional exposure between groups.

##### 2.3.2.1 Teaching materials

A standardized bank of 12 core quality and safety cases was developed, covering six domains: QC out-of-control events (*n* = 3), critical value delays (*n* = 2), patient identification errors (*n* = 2), specimen rejection scenarios (*n* = 2), result review discrepancies (*n* = 2), and adverse event near-misses (*n* = 1), with each case following a standardized template (Supplementary Material S1). In parallel, eight simulation scenarios were designed to address common communication and prioritization challenges, including critical value phone notification, specimen rejection communication with clinicians, same-name patient verification, and emergency specimen priority management, each accompanied by a structured evaluation checklist (Supplementary Material S2). These cases and scenarios were adapted across rotations to address dimension-specific teaching objectives. The overall integration of the three dimensions into routine teaching activities is summarized in Table 1.

**Table 1:** Integration of triple helix components into routine internship teaching.

Triple helix dimension	Conventional content	Triple helix–based teaching approach	Student activities	Key change
Quality Dimension	Traditional lectures on QC rules	Case-based discussion using standardized case bank, covering total process quality control across pre-analytical, analytical, and post-analytical phases, including specimen acceptance/rejection criteria, IQC interpretation, out-of-control management, critical value verification, and result review logic	Analyze authentic QC out-of-control events Trace root causes across total testing process Develop corrective action plans	Passive listening → Active analysis
Safety Dimension	Biosafety rule reviews	Scenario simulation and process drills integrating patient safety elements (patient identification, closed-loop critical value reporting, cross-position information handoff, adverse event/near-miss reporting, risk point identification) into foundational biosafety training	Participate in scenario-based drills Identify clinical consequences of safety lapses Practice closed-loop communication protocols	Rule memorization → Situational response
Patient-Centered Dimension	Routine result verification and technical skills training	Patient-centered integrated teaching using real-world scenarios (outpatient phlebotomy, specimen reception windows, critical value communication, clinical feedback tracking), focusing on pre-phlebotomy patient education, test explanation, special population care, and interdisciplinary collaboration	Result verification sessions: discuss how laboratory findings influence clinical decision-making Phlebotomy training: practice patient communication and test explanation	Technical skills → Technical + humanistic skills

##### 2.3.2.2 Faculty and training

Forty experienced preceptors implemented the triple helix–based intervention, following two 2-hour orientation sessions that covered hands-on strategies for weaving quality, safety, and patient-centered principles into daily teaching (e.g., quality case discussions, safety checklists in workflows, and communication simulations).

##### 2.3.2.3 Implementation substitutions

A blended teaching model was adopted, combining on-the-job immersion, case discussions, scenario simulations, reflective writing, and multi-preceptor collaborative mentoring. Three core substitutions were implemented within the existing rotation schedule: quality-focused (QC lectures replaced with case-based out-of-control analysis and corrective planning), safety-focused (biosafety reviews replaced with scenario-based drills on consequences and closed-loop communication), and patient-centered (result verification augmented with clinical impact case discussions). Rotation-specific adaptations are detailed in Table 2.

**Table 2:** Implementation of triple helix across subspecialty rotations.

Rotation	Quality dimension	Safety dimension	Patient-centered dimension
Clinical Chemistry	QC out-of-control case analysis; Westgard rule application	Closed-loop critical value reporting drills	Case discussions on how abnormal results impact clinical diagnosis and treatment
Clinical Immunology	Internal QC data trend analysis	Specimen handover and verification procedures	Communication and test explanation for special populations (e.g., autoimmune disease patients)
Clinical Microbiology	Specimen rejection criteria; antimicrobial susceptibility testing QC	Biosafety spill emergency drills	Clinical communication of microbiology reports and antimicrobial stewardship value
Clinical Hematology/Body Fluids	CBC/coagulation QC out-of-control management	Patient identification verification (same-name patient specimen handling)	Clinical communication of abnormal cell morphology reports
Molecular Diagnostics	PCR contamination prevention and QC	Genetic test result confidentiality and information transfer safety	Counseling communication points for genetic test results
Outpatient/Emergency Lab	STAT specimen prioritization and TAT management	**Critical** value immediate notification drills	Patient education at phlebotomy window; pre-analytical preparation communication

Dimension 1: Quality. Teaching covered total process quality control across pre-analytical, analytical, and post-analytical phases, including specimen acceptance/rejection, internal QC interpretation, out-of-control management, critical value verification, and result review logic. Content was delivered through case-based discussions using a standardized case bank, replacing conventional QC lectures. For example, in Clinical Chemistry, students analyzed QC out-of-control events using Westgard rules and traced root causes across the testing process; analogous case-based approaches were adapted across other rotations (see Table 2 for full details).

Dimension 2: Safety. Systematic safety content was integrated into foundational biosafety training, covering patient identification, closed-loop critical value reporting, cross-position handoffs, adverse event/near-miss reporting, and risk point identification. Scenario simulations and process drills using standardized scripts replaced routine biosafety rule reviews, emphasizing clinical consequences and closed-loop communication. For instance, Clinical Chemistry students practiced closed-loop critical value reporting drills; corresponding drills for each rotation are summarized in Table 2.

Dimension 3: Patient-Centered. Using real-world scenarios (phlebotomy, specimen reception, critical value communication, clinical feedback), training addressed pre-phlebotomy patient education, test explanation, special population care, and interdisciplinary collaboration. These elements were integrated into result verification and phlebotomy training rather than delivered as separate add-ons. In Clinical Chemistry, for example, case discussions centered on how abnormal results influence clinical decision-making; rotation-specific adaptations are presented in Table 2.

##### 2.3.2.4 Formative assessment activities

Reflective journals (one per rotation, six per student) were evaluated using a 5-domain rubric (problem identification, analysis depth, solution feasibility, patient-centered awareness, and self-reflection) on a 5-point Likert scale (Supplementary Material S3). Additionally, each student submitted one case tracking report for a representative clinical scenario, assessed using a standardized rubric (Supplementary Material S4).

##### 2.3.2.5 Implementation fidelity

Implementation fidelity was reinforced through monthly preceptor meetings and monitored via random observations of approximately 20% of teaching sessions, with completion logs maintained across all rotations (observation checklist and log template provided in Supplementary Materials S5–S6). Quantitative fidelity metrics were derived from these records: among 64 observed sessions (20% of all teaching encounters across the 48-week internship), 58 (90.6%) were rated as fully delivering the Triple Helix components according to the structured observation checklist; the remaining six sessions (9.4%) partially delivered the intended content due to time constraints or clinical urgency, with none rated as non-adherent. Completion logs confirmed nearly 100% attendance across all scheduled teaching activities. Collectively, these metrics indicate that the intervention was implemented with high fidelity, supporting the interpretability of the observed outcomes.

### 2.4 Evaluation indicators

At the end of each rotation, both groups underwent a series of comprehensive outcome assessments, including the Triple Helix Literacy Scale, Mini-Clinical Evaluation Exercise (Mini-CEX), process quality and safety indicators, written and practical skill examinations, and multi-source feedback, as detailed in the following subsections.

Outcomes were pre-specified as primary or secondary tiers based on their directness in reflecting the intervention's intended effects, aiming to maintain analytical clarity and minimize the risk of Type I error due to multiple comparisons.

#### 2.4.1 Primary outcomes

##### 2.4.1.1 Triple helix integrated literacy scale (thils) total score

This 36-item instrument measures interns' integrated competencies across quality, safety, and patient-centered literacy (12 items per dimension). Each item was scored on a 5-point Likert scale (1 = strongly disagree to 5 = strongly agree), with total scores ranging from 36 to 180. The scale was developed and validated through a systematic process including item generation, expert content validation, pilot testing, exploratory factor analysis, and test-retest reliability assessment; detailed psychometric properties are reported in Methods 2.6. Given the developmental stage of this instrument and the preliminary nature of its validation, its outcomes are classified as primary for the purpose of this exploratory study, but findings should be interpreted with appropriate caution.

##### 2.4.1.2 Mini-CEX comprehensive score

The five dimensions were adapted from the original seven competencies of the Mini-CEX framework to align with the study's focus on laboratory-integrated clinical practice (12, 13). The adaptation was conducted through a two-stage process. First, a panel of five experts (three senior laboratory educators and two clinical supervisors; >10 years' experience) reviewed the original domains, mapping each to laboratory medicine practice. History-taking and physical examination were excluded as they are not routinely performed by laboratory interns.

The remaining five domains—professionalism, communication skills, quality awareness, safety behaviors, and patient service—were retained, and behavioral anchors were reworded to reflect laboratory-specific contexts. Second, the adapted framework was pilot-tested with 10 preceptors, and refinements were made based on their feedback regarding clarity, feasibility, and relevance to routine teaching before full implementation. Content validity was established by the same expert panel, with I-CVI values ranging from 0.80 to 1.00 (S-CVI/Ave = 0.93).

Each domain was rated on a 9-point scale (1–3: unsatisfactory, 4–6: satisfactory, 7–9: superior). Behavioral anchors included, for example: professionalism—ranging from failure to demonstrate professional conduct to consistently exemplary behavior; quality awareness—ranging from failure to recognize quality issues to proactively identifying, reporting, and suggesting corrective actions; safety behavior—ranging from non-adherence to consistent adherence and promotion of safety protocols; communication skills—ranging from failure to convey test information clearly to patients and clinicians to effectively explaining test purposes, results, and follow-up recommendations; and patient service—ranging from lack of attention to patient comfort and needs to consistently demonstrating empathy, respect, and responsiveness during all patient interactions. The full behavioral anchor descriptors are provided in Supplementary Material S9.

Each intern underwent one assessment per clinical rotation (six encounters over 48 weeks). The composite score for each encounter was the mean of the five domain scores, and the overall score was the mean across all six encounters. Assessments were conducted by preceptors who completed a one-hour standardized training session on scoring criteria, case-based calibration, and common rating pitfalls. We explicitly note that assessors were not blinded to group allocation, and inter-rater reliability was not formally evaluated.

#### 2.4.2 Secondary outcomes

##### 2.4.2.1 Theoretical and practical skills examination scores

**Theoretical examination:** A standardized written examination covering core knowledge of laboratory medicine, quality management, and patient safety. Scores are reported on a **scale of 0 to 100** (with higher scores indicating better performance).

**Skills assessment:** An objective structured clinical examination (OSCE) or standardized operational assessment evaluating practical competencies including laboratory operating procedures, instrument use, quality control processes, and emergency response. Scores are also reported on a **scale of 0–100**.

##### 2.4.2.2 Process quality and safety indicators

Three indicators were retained. **QC out-of-control management accuracy** was defined as the percentage of events correctly identified and managed per protocols, derived from QC logs. **Critical value notification timeliness** was defined as the percentage of critical value notifications completed within 10 min from result verification to clinician notification, obtained from critical value notification records. **Patient identification compliance rate** was defined as the percentage of patient encounters where two independent identifiers (full name and date of birth) were verified prior to sample collection, assessed via direct observation audits.

These indicators were selected based on the following criteria: (i) each aligns with a distinct dimension of the Triple Helix framework; (ii) each has well-established operational definitions and standardized data collection procedures in routine laboratory practice; (iii) each is explicitly required or recommended by international laboratory standards (ISO 15189, CLSI guidelines); (iv) each has demonstrated sensitivity to educational interventions in previous studies; and (v) each is derived from existing quality management records, ensuring feasibility without additional measurement burden. The anticipated improvement (absolute risk difference) for each indicator was predefined as 15–25 percentage points, based on baseline performance levels observed in comparable trainee populations and effect sizes reported in prior quality improvement interventions.

For each intern, performance on each process indicator was aggregated as a pass/fail assessment based on multiple real-world observation opportunities across the 48-week internship. The minimum number of observations per intern was predefined for each indicator (≥5 for QC events, ≥3 for critical values, and ≥10 for patient identification audits). An intern was classified as “pass” if they met the predefined performance standard on ≥80% of observed opportunities; otherwise, they were classified as “fail”. Missing data was minimal (<5% across all indicators) and handled by complete-case analysis.

##### 2.4.2.3 Multi-source performance feedback (exploratory outcomes)

Multi-dimensional evaluations were collected from three stakeholder groups: **clinical staff** (ratings of interns' clinical performance), **patients** (ratings of interns' communication and service during phlebotomy or specimen collection), and **preceptors** (ratings of interns' overall learning engagement and competency development). Each rater provided a single global rating on the scale, with endpoints anchored at 0 (“very poor”) and 100 (“excellent”), based on their overall impression of the intern's performance in the respective domain. Scores were aggregated as the means of all available responses per intern on a 100-point visual analogue scale. Given the subjective nature of these measures and the absence of rater blinding, these outcomes are classified as exploratory.

### 2.5 Statistical analysis

Statistical analyses were performed using SPSS version 26.0 (IBM Corp., Armonk, NY, USA). Continuous data are presented as mean ± standard deviation (SD), with between-group comparisons conducted using independent-samples *t*-tests. Mean differences with 95% confidence intervals (CIs) and Cohen's d effect sizes are reported for continuous outcomes. Categorical data are expressed as frequencies and percentages. For 2 × 2 tables, Fisher's exact test was used when any expected cell count was <5; otherwise, the Pearson *χ*^2^-test was applied. For binary outcomes, risk differences with 95% CIs are reported. Consistent with the exploratory nature of this study, we did not prespecify a strict significance threshold. For primary outcomes, we elected not to apply multiple comparison corrections given the small number of pre-specified primary endpoints (two: THILS total score and Mini-CEX comprehensive score) and the exploratory intent of this study. For secondary and exploratory analyses, a Holm correction was applied to control the family-wise error rate; both adjusted and unadjusted *P*-values are reported for transparency. Owing to the non-contemporaneous cohort design and the small sample size, we placed greater emphasis on effect estimates (mean differences or risk differences) and their 95% CIs rather than on *P*-values alone. *P*-values are provided for descriptive purposes only and should not be interpreted as confirmatory evidence.

### 2.6 Development and validation of the triple helix integrated literacy scale

The Triple Helix Integrated Literacy Scale was developed as a primary outcome measure for this exploratory study. The development and validation process followed established methodologies for questionnaire design in health professions education (14).

#### 2.6.1 Scale development

An initial pool of 45 items was generated based on literature review of laboratory competency frameworks (ISO 15189, CLSI guidelines) and focus group discussions with 5 senior laboratory educators and 10 interns. Items were mapped to three hypothesized domains: quality literacy (15 items), safety literacy (15 items), and patient-centered service literacy (15 items).

#### 2.6.2 Content validity

A panel of 5 experts in laboratory medicine education rated each item for relevance on a 4-point scale (1 = not relevant to 4 = highly relevant). Item-level content validity indices (I-CVI) ranged from 0.80 to 1.00, and the scale-level content validity index based on average agreement (S-CVI/Ave) was 0.91.

#### 2.6.3 Pilot testing and item analysis

The preliminary 45-item scale was administered to a pilot sample of 15 interns (not included in the main study). Items with item-total correlation <0.30 were removed, resulting in a 36-item final version (12 items per dimension).

#### 2.6.4 Exploratory factor analysis

Exploratory factor analysis (EFA) was performed using principal axis factoring with Promax rotation on the pilot sample (*n* = 15). Three factors with eigenvalues >1.0 were extracted, collectively accounting for 62.4% of the total variance. Factor loadings ranged from 0.52 to 0.81, with all items loading onto their respective hypothesized domains.

#### 2.6.5 Test-retest reliability

The scale was readministered to the same pilot sample after a two-week interval. The intraclass correlation coefficient (ICC) was 0.84 (95% CI: 0.72–0.92), indicating good temporal stability.

#### 2.6.6 Scoring

Each item is rated on a 5-point Likert scale (1 = strongly disagree to 5 = strongly agree), with total scores ranging from 36 to 180. The full-scale questionnaire is provided in Supplementary Material S7.

#### 2.6.7 Internal consistency

In the main study sample (*n* = 62), the final 36-item scale demonstrated good internal consistency, with a Cronbach's *α* of 0.87.

#### 2.6.8 Criterion-related validity

The total scale score showed a moderate correlation with the Mini-CEX comprehensive score (*r* = 0.42, *P* < 0.01), providing preliminary evidence of criterion-related validity.

Collectively, these results provide preliminary psychometric evidence—including content validity, factor structure, internal consistency, and test-retest reliability—supporting the scale as a fit-for-purpose outcome measure in this exploratory study. However, given the small pilot sample (*n* = 15) on which the factor analysis was based, the factor structure should be interpreted with caution. The full scale and detailed validation results are provided in Supplementary Materials S7 and S8 to ensure transparency and to facilitate further validation of this instrument in larger, independent cohorts before broader application.

## 3 Results

### 3.1 Baseline characteristics

No statistically significant differences were found between the two groups in measured baseline characteristics, including age, sex distribution, and pre-internship theoretical and practical scores (all *P* > 0.05), indicating no obvious imbalance in the measured variables (Table 3).

**Table 3:** Baseline characteristics of the two groups.

Characteristic	Control Group (*n* = 30)	Experimental Group (*n* = 32)	Test Statistic^a^	P-value
Age (years)	22.13 ± 0.62	22.28 ± 0.58	*t* = 0.963	0.340
Gender (Male/Female)	11/19	12/20	*χ*^2^ = 0.005	0.946
Pre-internship theory score	78.63 ± 4.85	79.41 ± 5.12	*t* = 0.610	0.544
Pre-internship practical score	80.27 ± 5.04	81.19 ± 4.76	*t* = 0.739	0.463

a *t*-test for continuous variables.

χ^2^-test for categorical variables.

### 3.2 Primary outcomes: triple helix integrated literacy scale

Given the preliminary validation status of the Triple Helix Integrated Literacy Scale (see Methods 2.6), the following results should be interpreted as exploratory. The experimental group achieved higher scores than the control group across all three subdimensions—quality, safety, and patient-centeredness—as well as in the total score (all *P* < 0.001). The total score was (106.25 ± 7.96) in the experimental group versus (88.70 ± 8.25) in the control group, with a mean difference of 17.55 (95% CI: 13.42–21.66) and a large effect size (Cohen's d = 2.17), suggesting better internalization and integration of the three core competencies (Table 4). The final 36-item scale demonstrated good internal consistency in the current sample (Cronbach's *α* = 0.87, *n* = 62). Additional psychometric details are summarized in Supplementary Material S8.

**Table 4:** Comparison of triple helix integrated literacy scale scores between the two groups.

Dimension	Control group (*n* = 30)	Experimental group (*n* = 32)	Mean difference (95% CI)	Cohen's d	*P*-value
Quality dimension	30.17 ± 3.44	35.84 ± 3.12	5.67 (3.99–7.34)	1.73	<0.001
Safety dimension	29.63 ± 3.57	35.31 ± 3.26	5.68 (3.94–7.42)	1.66	<0.001
Patient-centered dimension	28.90 ± 3.76	35.09 ± 3.41	6.19 (4.36–8.02)	1.73	<0.001
Total score	88.70 ± 8.25	106.25 ± 7.96	17.55 (13.42–21.66)	2.17	<0.001

### 3.3 Primary outcomes: Mini-CEX scores

The experimental group showed higher scores than the control group across all five Mini-CEX domains (professionalism, communication skills, quality awareness, safety behaviors, and patient service) as well as in the overall composite score (all *P* < 0.001). The comprehensive score of the experimental group was 7.63 ± 0.53, compared to 6.47 ± 0.62 in the control group, with a mean difference of 1.16 (95% CI: 0.88–1.44) and a large effect size (Cohen's d = 2.02) (Table 5). The largest between-group differences were observed in the patient service domain (mean difference: 1.29, d = 1.78) and safety behaviors domain (mean difference: 1.22, d = 1.86). All interns completed the six scheduled Mini-CEX encounters, yielding a 100% completion rate.

**Table 5:** Comparison of mini-CEX scores between the two groups.

Domain	Control group (*n* = 30)	Experimental group (*n* = 32)	Mean difference (95% CI)	Cohen's d	*P*-value
Professionalism	6.73 ± 0.69	7.69 ± 0.64	0.96 (0.62–1.30)	1.44	<0.001
Communication skills	6.37 ± 0.81	7.50 ± 0.67	1.13 (0.76–1.50)	1.53	<0.001
Quality awareness	6.43 ± 0.77	7.63 ± 0.61	1.20 (0.86–1.54)	1.75	<0.001
Safety behaviors	6.50 ± 0.73	7.72 ± 0.58	1.22 (0.89–1.55)	1.86	<0.001
Patient service	6.30 ± 0.79	7.59 ± 0.66	1.29 (0.93–1.65)	1.78	<0.001
Comprehensive score	6.47 ± 0.62	7.63 ± 0.53	1.16 (0.88–1.44)	2.02	<0.001

### 3.4 Examination scores

The experimental group achieved higher theoretical examination scores (87.91 ± 4.98 vs. 82.47 ± 5.86), mean difference 5.44 (95% CI: 2.67–8.21), Cohen's d = 1.00 (*P* < 0.001), and skill assessment scores (90.28 ± 4.87 vs. 84.13 ± 6.02), mean difference 6.15 (95% CI: 3.36–8.94), Cohen's d = 1.13, than the control group (both *P* < 0.001) (Table 6).

**Table 6:** Comparison of final examination scores between the two groups.

Examination category	Control group (*n* = 30)	Experimental group (*n* = 32)	Mean difference (95% CI)	Cohen's d	*P*-value
Theoretical examination score	82.47 ± 5.86	87.91 ± 4.98	5.44 (2.67–8.21)	1.00	<0.001
Skill assessment score	84.13 ± 6.02	90.28 ± 4.87	6.15 (3.36–8.94)	1.13	<0.001

### 3.5 Process quality and safety indicators

The experimental group showed a higher accuracy rate in quality control out-of-control management compared with the control group (93.8% vs. 70.0%; risk difference 23.75%, 95% CI: 5.33–42.17; *χ*^2^ = 6.04, *P* = 0.014) and a higher timeliness rate of critical value notification (100% vs. 80.0%; risk difference 20.00%, 95% CI: 5.69–34.31; Fisher's exact test, *P* = 0.010). The patient identification compliance rate was also higher in the experimental group than in the control group (93.8% vs. 73.3%; risk difference 20.42%, 95% CI: 2.51–38.33; Fisher's exact test, *P* = 0.040). Following Holm correction for multiple comparisons, the adjusted *P*-values were 0.028 for QC out-of-control management accuracy, 0.030 for critical value notification timeliness, and 0.040 for patient identification compliance rate (Table 7).

**Table 7:** Comparison of process quality and safety indicators between the two groups.

Indicator	Control group (*n* = 30)	Experimental group (*n* = 32)	Risk difference (95% CI)	Test statistic^†^	*P*-value
QC out-of-control management accuracy	21 (70.0)	30 (93.8)	23.75 (5.33–42.17)	χ^2^ = 6.04	0.014
Critical value notification timeliness	24 (80.0)	32 (100.0)	20.00 (5.69–34.31)	Fisher's exact	0.010
Patient identification compliance rate	22 (73.3)	30 (93.8)	20.42 (2.51–38.33)	Fisher's exact	0.040

Data are presented as *n* (%). Risk differences are presented as percentage points (95% CI). ^†^Fisher's exact test was used for comparisons in which any expected cell count was < 5 (i.e., critical value notification timeliness and patient identification compliance rate); Pearson's chi-square test was applied for QC out-of-control management accuracy.

### 3.6 Multi-stakeholder satisfaction (exploratory outcomes)

The experimental group received higher performance ratings from **clinical staff** (92.88 ± 4.36 vs. 86.43 ± 5.91)**,** mean difference 6.45 (95% CI: 3.80–9.10)**, patients** (91.34 ± 4.97 vs. 84.77 ± 6.18)**,** mean difference 6.57 (95% CI: 3.71–9.43), and **preceptors** (92.41 ± 4.26 vs. 87.03 ± 5.43)**,** mean difference 5.38 (95% CI: 2.89–7.87) than the control group (all *P* < 0.001) (Table 8).

**Table 8:** Multi-source performance feedback by stakeholder group.

Stakeholder	Control group (*n* = 30)	Experimental group (*n* = 32)	Mean difference (95% CI)	Cohen's d	*P*-value
Clinical staff	86.43 ± 5.91	92.88 ± 4.36	6.45 (3.80–9.10)	1.25	<0.001
Patients	84.77 ± 6.18	91.34 ± 4.97	6.57 (3.71–9.43)	1.18	<0.001
Preceptors	87.03 ± 5.43	92.41 ± 4.26	5.38 (2.89–7.87)	1.11	<0.001

## 4 Discussion

The clinical internship is critical for professional competency development (6). Conventional teaching emphasizes technical skills but inadequately addresses quality management, systematic safety, and patient-centered care. The Triple Helix framework—quality as foundation, safety as bottom line, and patient-centeredness as guiding principle—was designed to restructure internship training to address these gaps.

Our findings align with previously reported single-dimensional reforms while extending them in important ways. Zhang et al. (6) reported that competency-based training improved interns' technical proficiency, but their evaluation did not assess patient-centered or systematic safety outcomes. Similarly, Xie et al. (15) demonstrated that modular teaching enhanced hematology performance, yet their evaluation focused on discipline-specific knowledge rather than integrated competencies. Ma et al. (16) reported the concept of integrative experimental teaching in basic clinical laboratory medicine, suggesting potential advantages over traditional teaching; however, their integration remained confined to horizontal alignment of experimental content within a single course rather than vertical integration across multiple competency dimensions throughout the entire internship curriculum. More broadly, while quality-focused interventions have emphasized total quality management and Six Sigma methodologies, and safety-focused reforms have addressed occupational biosafety and infection control, to our knowledge, few published studies have systematically integrated quality, safety, and patient-centeredness into a unified training framework for laboratory interns.

In contrast, our triple helix model extends the integrative concept vertically across three core dimensions—quality, safety, and patient-centeredness—spanning the full 48-week internship rather than a single course. Notably, the experimental group showed concurrent improvements across all three domains, suggesting that the synergistic design may achieve broader competency development than single-dimensional interventions. The effect sizes observed (Cohen's d ranging from 1.00 to 2.17) are generally comparable to, and in some cases tend toward the upper end of, those reported in similar educational interventions in health professions education (17). However, given the exploratory design and cross-study heterogeneity in outcome measures, these comparisons are preliminary and hypothesis-generating rather than confirmatory.

The quality dimension training strengthens interns' total process laboratory thinking. Modern laboratory practice demands not only proficient execution of testing workflows but also the ability to identify and manage factors affecting result reliability across the total testing process. ISO 15189 emphasizes total process quality management from specimen collection, transportation, reception, and testing through to result reporting (18). In this study, the experimental group's better performance in QC out-of-control management accuracy suggests that quality case discussions, out-of-control event management training, and result review logic analysis facilitate students' transition from merely mastering operational procedures to understanding quality control mechanisms. However, the improvement in QC out-of-control management accuracy and critical value notification timeliness cannot be attributed solely to case discussions or simulation training. Rather, we suggest that the triple helix synergy operates through three mutually reinforcing layers.

First, the **quality dimension** provided the cognitive foundation. Case-based discussions trained interns to systematically interrogate QC failures, tracing root causes across pre-analytical, analytical, and post-analytical phases. For example, interns analyzing Westgard rule violations (Table 2) were required to propose corrective actions across specimen integrity, calibration, and result verification—cultivating systematic thinking. This analytical habit—moving from error identification to systemic cause analysis—represents a key component of systematic thinking.

Second, the **safety dimension** supplied the risk-awareness amplifier. Scenario-based drills concretized the clinical consequences of quality lapses—delayed critical values, misidentification, and information handoff failures—deepening interns' appreciation of why quality verification matters beyond technical compliance. As shown in Table 1, these drills replaced passive biosafety rule reviews with active situational responses, requiring interns to practice closed-loop communication protocols for critical value notification.

Third, the **patient-centered dimension** furnished the motivational anchor. Reflective exercises and case tracking connect abstract quality indicators and safety protocols to tangible patient outcomes, fostering internalization of professional values. For example, during phlebotomy training, interns practiced structured patient communication—including two-identifier verification, test explanation, and post-collection care guidance—operationalizing patient-centeredness as a daily practice rather than abstraction. This integration of technical skills with humanistic skills (Table 1) fosters internalization of professional values.

The concurrent improvements observed across the three outcome domains are consistent with—but do not prove—the proposed synergistic mechanism. The triple helix logic posits that quality, safety, and patient-centeredness may reinforce one another through shared cognitive and motivational pathways. For example, quality case discussions may sharpen analytical habits, safety drills may concretize the clinical consequences of quality lapses, and patient-centered reflection may anchor both in tangible patient outcomes. However, the present study was designed as a global group-level comparison, not a factorial experiment with component-isolated arms. Therefore, we cannot disentangle the unique contribution of each dimension or confirm interactive effects among them. Whether the observed improvements reflect true synergy, additive effects of three independent components, or merely the cumulative benefit of increased teaching attention remains an open question. Accordingly, the synergy hypothesis should be regarded as a theoretical lens for interpreting the results and generating future research questions, rather than as a demonstrated mechanism.

The safety dimension illustrates how this synergistic logic translates into measurable improvements. Clinical laboratory work is closely tied to patient safety—errors such as delayed critical values, misidentification, specimen mix-ups, and information gaps can all lead to serious adverse events. Conventional safety training, however, focuses narrowly on occupational biosafety, with limited systematic instruction across the total testing process, leaving students with insufficient understanding of how their daily operations affect clinical outcomes. In this study, the experimental group's higher critical value notification timeliness suggests that scenario-based training—including critical value reporting, same-name patient verification, and emergency specimen prioritization—may facilitate the development of students' ability to identify and respond to systematic safety risks. The improvement in patient identification compliance rates reached only borderline significance after adjustment, warranting cautious interpretation and suggesting that further reinforcement through scenario-based exercises and process feedback may still be needed. Collectively, these findings appear to support expanding the concept of “safety” from personal protection to encompass patient safety, process safety, and information security, potentially contributing to a total process-oriented risk prevention mindset and encouraging the cognitive shift from “I completed the test” to “I ensured the right patient received the right result at the right time.”

Beyond safety, the third helix—patient-centeredness—addresses a different but equally critical challenge: how to translate professional obligations into concrete behaviors despite limited direct patient contact. The inherent constraints of laboratory medicine on direct patient contact do not exempt laboratory professionals from their patient-centered responsibilities. Drawing on ISO 15189 patient-centered clauses, we propose embedding three standardized workflows into daily rotation practice to translate the patient-centered concept into concrete, teachable behaviors. First, **structured phlebotomy communication** can be implemented at outpatient collection windows, incorporating a standardized script covering: (i) two-identifier patient verification (full name and date of birth); (ii) test explanation and pre-collection instructions; (iii) post-collection care guidance; and (iv) expected turnaround time communication. Second, **closed-loop critical value notification** can be formalized with a “read-back and verify” protocol requiring the receiving clinician to repeat the critical result verbatim, with documentation of notification time and recipient identity, followed by 24-hour clinical response tracking. Third, **special population service adaptations** can be developed for pediatric, geriatric, and obstetric patients, including age-appropriate communication strategies and differentiated care considerations. Importantly, these workflows do not require additional teaching time; they are embedded into existing clinical activities—phlebotomy shifts, specimen reception, and result verification—and serve as natural teaching moments during routine supervision. We recommend that each intern complete at least two structured communication encounters per rotation with direct preceptor observation and feedback, operationalizing patient-centeredness as a daily practice rather than abstraction. This approach helps students recognize that although laboratory work involves limited direct patient contact, every verification, every communication, and every report directly impact patient welfare and healthcare quality. “Patient-centeredness” does not require laboratory professionals to assume the full communication responsibilities of clinicians; rather, it requires them to perform within their scope of duty—accurate verification, clear explanation, timely feedback, and respectful care.

Beyond reshaping teaching content, our approach also redesigned how interns' performance was evaluated. Unlike traditional methods relying solely on final theoretical and skill examinations, our multidimensional system incorporated process indicators, Mini-CEX, literacy scales, and multi-stakeholder satisfaction surveys. This system more accurately reflects students' normative practice, safety performance, and service capabilities in real clinical settings, and aligns better with the current trends in formative assessment and competency-based evaluation in medical education (19). The experimental group's favorable results across multiple dimensions suggest that the multidimensional evaluation system not only facilitates comprehensive assessment of teaching effectiveness but also provides reverse guidance for shifting teaching priorities from purely technical training toward integrated competency development.

For tertiary hospital laboratories, this framework aligns with ISO 15189 standards, patient safety goals, and quality requirements. Importantly, our findings are consistent with the reform directions recently advocated in the domestic context, where multidimensional integration of teaching systems has been emphasized as a key strategy for laboratory internship reform under the New Medicine framework (20).

## 5 Limitations

Despite these observations, several limitations should be acknowledged.

### 5.1 Design and confounding

Several limitations pertaining to study design and confounding warrant consideration. **First**, the non-contemporaneous historical control design precludes causal inference. In the absence of baseline measurements for the principal competency outcomes—the Mini-CEX, safety behaviors, patient-service skills, and integrated literacy—pre-existing differences between the 2022 and 2023 cohorts cannot be ruled out. Although demographic and academic characteristics were comparable across groups, unmeasured cohort-specific attributes (e.g., intrinsic learning motivation, prior clinical exposure) may have contributed to the observed post-intervention differences. **Second,** this design is vulnerable to temporal confounding. Time-varying factors, including fluctuations in clinical workload, institutional quality campaigns, and preceptor experience, may have systematically differed between the two years and either inflated or attenuated the effect estimates. **Third,** because the Triple Helix framework was integrated into routine clinical training rather than delivered as a standalone module, isolating its specific contribution from other components of the internship experience remains inherently challenging. **Nevertheless**, the magnitude and consistency of the observed effects across multiple outcome domains—together with the fact that any historical contamination would have biased results toward the null—suggest that the intervention likely contributed meaningfully to the observed improvements, even though the precise effect size cannot be definitively established. Collectively, these constraints indicate that our findings should be interpreted as preliminary associations rather than evidence of causal effectiveness.

### 5.2 Blinding and assessment bias

Assessors were not blinded to group allocation and also delivered the intervention, introducing potential expectation and observer bias. Patient and preceptor performance ratings are subjective and susceptible to social desirability bias. Furthermore, the non-independence arising from multiple ratings per intern and ratings clustered by preceptor was not adjusted for in the analysis.

### 5.3 Sample size and generalizability

The small sample (*n* = 62) from a single tertiary teaching hospital limits statistical power and generalizability to primary care laboratories, non-degree training programs, or different geographic settings. The lack of *a priori* sample size calculation limits the precision of effect estimates, although the observed differences were consistently large across outcomes.

### 5.4 Outcome measurement

Several outcome measurement limitations should be considered. **First,** THILS was developed specifically for this exploratory study. Its structural validity is preliminary, as the exploratory factor analysis was based on a small pilot sample (*n* = 15) and has not been cross-validated independently. Therefore, THILS scores should be interpreted as exploratory indicators requiring further psychometric evaluation. **Second,** for the Mini-CEX, the absence of formal inter-rater reliability testing and the non-blinded assessment design represent significant validity threats, as the subjective nature of these ratings may have systematically inflated the experimental group's scores through observer and expectation biases. **Third**, the concurrent administration of the THILS and Mini-CEX introduces potential common method bias, as both were completed by the same preceptors who delivered the intervention. **Fourth,** although the three process indicators were derived from objective records, their relatively high baseline performance—particularly critical value notification timeliness (80.0% in the control group)—raises ceiling effect concerns that may limit sensitivity for detecting incremental improvements and constrain generalizability to less well-resourced laboratories with more room for improvement.

### 5.5 Statistical interpretation warrants caution

Although the number of primary endpoints was limited (two), the absence of multiple comparison adjustment for these outcomes may increase the risk of Type I error; these findings should therefore be interpreted as exploratory. Furthermore, the observed Cohen's d effect sizes were large (range: 1.00–2.17). While these values suggest substantial group differences, the non-blinded assessment design may have systematically inflated the estimates, particularly for subjective measures. Nevertheless, the objectively measured outcomes—theoretical and practical examination scores—also demonstrated meaningful differences (Cohen's d = 1.00 and 1.13, respectively), suggesting that the observed effects are unlikely to be attributable solely to assessment bias. However, replication in rigorously designed studies with blinded assessments is essential to obtain unbiased effect estimates.

### 5.6 Long-term outcomes

This study assessed outcomes only at the time of internship completion. No data are available on post-graduation workplace performance, error rates, clinical consultation behaviors, knowledge and skills retention during subsequent residency or employment, or the durability of the observed competency gains. Whether the improvements associated with the Triple Helix model translate into sustained practice improvements or better patient outcomes beyond the internship period remains unknown. Future studies with extended follow-up are needed to assess long-term effectiveness.

Nonetheless, the findings provide preliminary evidence that the multidimensional integrated teaching model is feasible and associated with favorable educational outcomes.

## 6 Conclusion

In this exploratory quasi-experimental study, implementation of the triple helix model was associated with better end-of-internship performance across knowledge, skills, quality awareness, safety behaviors, patient-centered competencies, and satisfaction. These preliminary findings suggest that the integrated framework may address key shortcomings of traditional training by supporting concurrent development across quality, safety, and patient-centered domains while maintaining foundational technical instruction.

Nevertheless, the proposed synergistic mechanism among the three Triple Helix dimensions remains speculative. The global group-level comparison does not permit isolation of each component's unique contribution or formal testing of interactive effects. Without component-isolated experimental arms, we cannot determine whether the observed improvements reflect true synergy, additive effects, or nonspecific benefits of increased instructional attention.

Moreover, causal inference is constrained by the non-randomized historical control design. Future research should therefore employ randomized or quasi-experimental designs with blinded assessment, formal inter-rater reliability testing, independent validation of the Triple Helix Integrated Literacy Scale in larger and more diverse populations, **factorial or component-wise designs to disentangle individual and interactive effects**, objective long-term performance measures, and implementation across varied laboratory settings to corroborate these findings and evaluate the replicability of this framework.

## Data Availability

The raw data supporting the conclusions of this article will be made available by the authors, without undue reservation.
