# Supplementary material for: Development and evaluation of a practical teaching model for medical laboratory interns based on the “quality-safety-patient-centered” triple helix framework
Source: Front Med (Lausanne). 2026 Oct 14;13:1936253. doi: 10.3389/fmed.2026.1936253 (PMC13713319; doi:10.3389/fmed.2026.1936253)
Supplement: Supplementary file 1 [file Datasheet1.pdf]

Supplementary Materials Index

S1. Core Quality and Safety Standardized Case Bank for the "Quality-Safety-Patient-Centered" Triple Helix Teaching Model……………………………………………1

S2. Quality-Safety-Patient-Centered" Triple Helix Teaching Model — Scenario-Based Simulation Training Scenarios and Assessment Tools…………………………26

S3. Reflective Journal Scoring Rubric (5-point Likert Scale) ……………………….49

S4. Case Tracking Report Scoring Rubric (Standardized Rubric) …………………. 51

S5. Observation Checklist for Implementation Fidelity of Triple Helix Teaching Sessions……………………………………………………………………52

S6. Teaching Session Attendance and Completion Log……………………………..54

S7. Triple Helix Integrated Literacy Scale (THILS)…………………………………56

S8. Psychometric Properties of the Triple Helix Integrated Literacy Scale…………..61

S9. Mini-CEX Domains and 9-Point Behavioral Anchors…………………………....62

Supplementary Material S1:

Core Quality and Safety Standardized Case Bank for the "Quality-Safety-Patient-Centered" Triple Helix Teaching Model

1. Overview of Case Categories and Distribution

Case Category	Case IDs	Count	Primary Rotation
QC Out-of-Control Events	C1–C3	3	Clinical Chemistry, Clinical Hematology, Clinical Immunology
Critical Value Delays/Communication Failures	C4–C5	2	Outpatient/Emergency Lab, Clinical Chemistry
Patient Identification Errors	C6–C7	2	Clinical Hematology/Body Fluids, Molecular Diagnostics
Specimen Rejection Scenarios	C8–C9	2	Clinical Microbiology, Outpatient/Emergency Lab
Result Review Discrepancies/Reporting Errors	C10–C11	2	Clinical Chemistry, Molecular Diagnostics
Adverse Event/Near-Miss	C12	1	Clinical Immunology
**Total**		**12**	

2. Case Template Description

Each case is presented using a standardized template with the following fields:

Field	Description
Case ID	Unique identifier (C1–C12)
Rotation	Primary laboratory rotation in which the case is used
Core Topic	Central quality/safety issue addressed
Scenario	Narrative description of the clinical/laboratory situation
Triple Helix Dimensions	Quality ☐ / Safety ☐ / Patient-Centered ☐ (checked as applicable)
Learning Objectives	2–3 specific, measurable objectives (student-oriented)
Teaching Points	Key content for preceptors to emphasize during discussion
Discussion Questions	2–3 guiding questions for student discussion
Suggested Format	Recommended teaching format and duration

Note: Learning objectives are student-oriented statements of what students will be able to do after the case; teaching points are preceptor-oriented guidance on key content to emphasize during discussion.

3. Detailed Case Descriptions

Category 1: QC Out-of-Control Events

Case C1: Clinical Chemistry — Westgard 1_{3s} Rule Violation

Field	Content
**Case ID**	C1
**Rotation**	Clinical Chemistry
**Core Topic**	Identification and management of internal QC out-of-control events
**Scenario**	On a weekday morning, the Clinical Chemistry laboratory detected a QC violation for creatinine (Cr) during routine patient testing. The high-level QC result exceeded the +3s limit (Westgard 1_{3s} rule triggered). A total of 47 patient results for creatinine had been released prior to detection of the violation.
**Triple Helix Dimensions**	☑ Quality ☑ Safety ☐ Patient-Centered
**Learning Objectives**	1. Correctly identify a Westgard 1_{3s} out-of-control signal and understand its clinical significance;<br>2. Execute the standard out-of-control management protocol (Stop → Report → Investigate → Retest → Document);<br>3. Assess the potential impact on patient safety and formulate a recall/retest plan for previously released reports.
**Teaching Points**	• Clinical application of Westgard multirules and the meaning of the 1_{3s} rule (random error);<br>• Systematic troubleshooting sequence: reagent → calibrator → QC material → instrument → operator technique;<br>• Criteria for determining the scope of report recall/retesting;<br>• Communication strategies and scripts for notifying clinicians of corrected results.
**Discussion Questions**	1. What are the immediate actions the technologist should take upon triggering a 1_{3s} rule?<br>2. How would you determine which of the 47 released reports need to be recalled or retested?

Field	Content
	3. If retesting confirms that some reports are indeed biased, how should you communicate with the clinical team?
**Suggested Format**	Morning report case discussion + decision-making simulation (30 minutes)

Case C2: Clinical Hematology — Coagulation QC Trend Shift

Field	Content
**Case ID**	C2
**Rotation**	Clinical Hematology
**Core Topic**	Identification and proactive intervention of QC trend shifts
**Scenario**	Internal QC results for coagulation testing (PT, APTT) showed a unidirectional shift for five consecutive days—all trending in the same direction away from the target mean. However, all daily results remained within the ±2s range and did not trigger any out-of-control rules. The preceptor noted the abnormal trend, but some colleagues argued that "as long as it's in control, no intervention is needed."
**Triple Helix Dimensions**	☑ Quality ☑ Safety ☐ Patient-Centered
**Learning Objectives**	1. Recognize the warning significance of trend shifts (five consecutive unidirectional shifts) on Levey-Jennings charts;<br>2. Understand the potential clinical risks of "in-control but drifting" QC patterns;<br>3. Develop proactive intervention strategies (e.g., reagent lot change, recalibration).
**Teaching Points**	• Interpretation of trend patterns on Levey-Jennings charts;<br>• Distinction between shift, trend, and out-of-control events—and intervention thresholds (application of Westgard 2_{2s} and 10x rules);<br>• QC transition management when changing reagent lots;

Field	Content
	• Clinical impact of QC drift on warfarin and other anticoagulant dosing decisions.
**Discussion Questions**	1. Why is "in-control but drifting" more insidious than a single out-of-control event?<br>2. How would you determine whether the drift originates from reagents, calibrators, or the instrument?<br>3. Should testing be proactively paused even when no out-of-control rule has been triggered? What is your rationale?
**Suggested Format**	Levey-Jennings chart interpretation exercise + group discussion (20 minutes)

Case C3: Clinical Immunology — ELISA R_{4s} Rule Violation

Field	Content
**Case ID**	C3
**Rotation**	Clinical Immunology
**Core Topic**	Multi-rule interpretation and systematic troubleshooting
**Scenario**	During a batch run of ELISA testing for hepatitis B markers (HBsAg, anti-HBs, HBeAg, anti-HBe, anti-HBc), two levels of QC materials yielded results at +4s and −2s respectively within the same run, triggering the R_{4s} rule (indicating random error). A total of 32 clinical specimens had been tested and reported in this batch.
**Triple Helix Dimensions**	☑ Quality ☑ Safety ☐ Patient-Centered
**Learning Objectives**	1. Understand the meaning of the R_{4s} rule and the type of error it indicates (random error);<br>2. Systematically identify common sources of random error in ELISA testing;<br>3. Determine the necessity and scope of batch retesting.

Field	Content
**Teaching Points**	• Distinction between R_4s and 1_3s rules (random error vs. systematic error);<br>• Common sources of random error in ELISA: pipetting error, uneven incubation temperature, incomplete washing, well-to-well variation in plate readers;<br>• Principles for determining retesting scope and communication considerations with clinicians;<br>• Clinical weight of hepatitis B marker reports (e.g., impact of false-negative HBsAg on blood transfusion safety).
**Discussion Questions**	1. What should be the sequence of troubleshooting upon triggering an R_4s rule?<br>2. Which ELISA steps are most prone to introducing random error, and how can they be prevented?<br>3. Among the 32 released reports, which patients' results should be prioritized for retesting?
**Suggested Format**	Case discussion + hands-on troubleshooting exercise (30 minutes)

Category 2: Critical Value Delays/Communication Failures

Case C4: Outpatient/Emergency Lab — STAT Cardiac Marker TAT Delay and Critical Value Notification

Field	Content
**Case ID**	C4
**Rotation**	Outpatient/Emergency Laboratory
**Core Topic**	STAT specimen prioritization, total TAT monitoring, and critical value notification
**Scenario**	A high-sensitivity cardiac troponin T (hs-cTnT) specimen — collected from a routine Emergency Department (ED) patient (non-STEMI pathway) rather than from the Chest Pain Center — was obtained at 09:15 and received by the emergency laboratory. Owing to a surge in routine outpatient specimens, the sample was neither flagged nor prioritized as STAT, resulting in verification being delayed until 10:25, at which point a critical result of 0.52 μg/L was reported. The technologist immediately notified the ED; however, the attending physician was engaged in a resuscitation and the critical value was consequently relayed via nursing staff, with notification documented at 10:28 (within 3 minutes of verification). Despite this, the physician did not formally acknowledge the result until 10:45, during shift handover. The total turnaround time (TAT) from collection to verification was 70 minutes — 10 minutes beyond the 60-minute STAT benchmark. While notification timeliness was compliant (≤10 minutes), the indirect communication pathway via nursing staff delayed both clinician awareness and subsequent therapeutic decision-making.
**Triple Helix Dimensions**	☑ Quality ☑ Safety ☑ Patient-Centered
**Learning Objectives**	1. Understand the necessity of STAT specimen prioritization, analyze root causes of TAT delays, and propose improvement measures;<br>2. Master the critical value notification timeliness standard (≤10 minutes from result verification to clinician notification) and

Field	Content
	recognize that closed-loop communication requires direct clinician receipt and read-back, not merely documented notification;<br>3. Explore the impact of delayed testing and delayed clinical response on outcomes in acute coronary syndrome (ACS) patients.
**Teaching Points**	• "Routine ED" is not synonymous with "STAT" — automatic STAT designation typically applies to Chest Pain Center/STEMI pathways, Code Blue, or explicitly ordered urgent tests; routine ED specimens follow standard processing queues unless manually flagged;<br>• STAT specimen identification and processing protocols— pre-analytical flagging and LIS integration;<br>• Total TAT standards (≤60 min from collection to verification/ report) vs. notification timeliness standards (≤10 min from verification to clinician notification)—distinct but complementary;<br>• "Notification documented" ≠ "clinician awareness" — direct receipt and read-back are required for closed-loop communication;<br>• Tiered triage strategies during specimen backlog (e.g., STAT vs. routine, Chest Pain Center vs. general ED);<br>• "Time is myocardium"—ischemic time and irreversible injury;<br>• Common notification chain failures: delayed verification, delayed call placement, clinician unreachable, message relay without direct receipt, incomplete documentation;<br>• Root cause analysis (RCA) for TAT delays and communication breakdowns;<br>• Legal and ethical risks of delayed testing and delayed clinical response.
**Discussion Questions**	1. At which steps (pre-analytical, analytical, post-analytical) did the total TAT delay occur, and how could each be optimized?<br>2. The notification call was completed within 3 minutes (meeting the ≤10-min standard), but the physician did not act until 20 minutes later due to message relay. Was the notification process successful? What else could the technologist have done to close the loop?<br>3. How would you balance STAT vs. routine specimens during backlog?<br>4. How would you distinguish between "notification timeliness" (laboratory-controlled) and "clinical response timeliness" (system-level) in quality improvement?

Field	Content
	5. How would you communicate the delay to the clinician and mitigate patient impact?<br>6. How would you redesign the critical value notification process to prevent relay breakdowns during physician unavailability (e.g., resuscitation, procedures)? What alternatives exist beyond "leave a message with the nurse"?
**Suggested Format**	Scenario simulation + root cause analysis (RCA) + critical value notification role-play (40 minutes)

Case C5: Clinical Chemistry — Unclosed Critical Value Call

Field	Content
**Case ID**	C5
**Rotation**	Clinical Chemistry
**Core Topic**	Closed-loop critical value reporting integrity
**Scenario**	The on-duty technologist detected a critical value for serum potassium (K^+ 6.8 mmol/L) and called the Nephrology ward nursing station per protocol. The nurse who answered the phone read back "potassium 6.8." However, the technologist did not require the nurse to read back the patient's name and hospital number, nor did he record the caller's staff ID or the ward. After the call, the technologist marked "notified" in the LIS. Upon follow-up the next day, it was discovered that the critical value had not been recorded in the nursing shift handover log or the nursing record, posing a potential risk that the patient did not receive timely intervention.
**Triple Helix Dimensions**	☑ Quality ☑ Safety ☑ Patient-Centered

Field	Content
**Learning Objectives**	1. Master the standard closed-loop critical value reporting process (Notify → Read-back → Confirm → Document);<br>2. Understand the mechanism by which broken communication chains impact patient safety;<br>3. Practice standardized critical value call scripts and response techniques.
**Teaching Points**	• Six essential elements of closed-loop critical value reporting: patient name, hospital number, test name, result value, reference range, and recipient identification;<br>• Dual requirements of "read-back" and "documentation";<br>• ISO15189 requirements for critical value reporting;<br>• Standardized script template (SBAR: Situation-Background-Assessment-Recommendation);<br>• Legal precedents of patient harm due to broken communication chains.
**Discussion Questions**	1. At which step did the closed loop break in this case?<br>2. How would you design a standardized script to ensure completeness of every critical value call?<br>3. What should you do if the recipient refuses to provide their name or staff ID?
**Suggested Format**	Role-play + script practice (20 minutes)

Category 3: Patient Identification Errors

Case C6: Clinical Hematology/Body Fluids — Same-Name Patient Specimen Mix-up

Field	Content
**Case ID**	C6
**Rotation**	Clinical Hematology/Body Fluids
**Core Topic**	Dual patient identification and specimen labeling management
**Scenario**	Two male patients with identical names ("Wang Wei," aged 52 and 67) were admitted to different rooms within the same ward of the inpatient department. The responsible nurse verified only bed number and name—without using the unique identifier (hospital number)—during morning phlebotomy, and applied the bar-coded labels to the wrong specimens. The laboratory did not detect the error during specimen receipt or testing, and both CBC reports were released under the incorrect patient identities. The next day, the clinician noted that Patient A's (age 52) hemoglobin had dropped from 130 g/L to 82 g/L—clinically inconsistent with his condition—and tracing confirmed specimen misidentification.
**Triple Helix Dimensions**	☑ Quality ☑ Safety ☑ Patient-Centered
**Learning Objectives**	1. Establish a mandatory habit of dual patient identification (name + hospital number/date of birth);<br>2. Identify the "critical control points" for patient identification throughout the testing workflow;<br>3. Develop systematic strategies to prevent specimen mix-ups for same-name patients.
**Teaching Points**	• Application of the "three checks and seven verifications" principle in laboratory settings;<br>• Identification barriers at the specimen receipt stage (barcode scanning, LIS auto-verification);<br>• System-level solutions: electronic wristbands, PDA scanning, dual manual verification;<br>• Traceback protocol (from released report → original specimen →

Field	Content
	phlebotomy step);<br>• Recall and correction procedures for erroneous reports already released to clinicians.
**Discussion Questions**	1. Which step in this case should have caught the error? Why did it fail?<br>2. If the laboratory has no authority to change clinical phlebotomy procedures, how could you advocate for systemic improvement?<br>3. How would you initiate the recall process if the erroneous reports have already been released?
**Suggested Format**	Case discussion + workflow redesign exercise (30 minutes)

Case C7: Molecular Diagnostics — Well-Recording Error During Nucleic Acid Extraction Leading to EGFR Result Misassignment

Field	Content
**Case ID**	C7
**Rotation**	Molecular Diagnostics
**Core Topic**	Manual recording errors during nucleic acid extraction leading to breakdown of specimen identity traceability
**Scenario**	A molecular lab received peripheral blood EGFR test specimens from two lung adenocarcinoma patients (Patient A, 58, and Patient B, 64) on the same day. Labels and LIS entries were correct. Both specimens were placed in adjacent wells (A1 for Patient A, B1 for Patient B) on the same extraction plate. During manual recording of the well layout on a paper log sheet, the technologist reversed the patient names for the two wells. This error carried through to PCR tube labeling and result association, leading to a cross-assignment of the two patients' EGFR results. During result review, the reviewer noted that both results were inconsistent with each patient's prior testing history —Patient A, who had a recent negative result, now showed a positive result (inconsistent); Patient B, who was

Field	Content
	previously positive and responding to therapy, now showed a negative result (contradictory). These discrepancies prompted the reviewer to hold the reports. Trace-back confirmed that the well-recording error had caused the misassignment. The error was corrected before release, and neither patient was affected.
**Triple Helix Dimensions**	☑ Quality ☑ Safety ☑ Patient-Centered
**Learning Objectives**	1. Understand the requirements for maintaining the "identity chain" throughout the entire molecular diagnostic workflow — from specimen tube → extraction plate → amplification → final report;<br>2. Recognize the risk of identity correspondence breakdown during manual well-recording in the nucleic acid extraction step;<br>3. Master standardized methods for manual recording and understand electronic alternatives;<br>4. Establish the quality awareness that "the review step is the last line of defense."
**Teaching Points**	• **Key nodes of the identity chain:** Specimen tube → extraction plate (well assignment) → PCR tube (transfer) → instrument (result linkage) → LIS (report). A break at any node can cause misassignment. In this case, the break occurred at the "extraction plate well assignment → PCR tube transfer" step.<br>• **Risks of manual recording:** Handwritten entries are error-prone, illegible, and difficult to trace. The risk increases significantly when multiple specimens are processed together.<br>• **Recording principles:** Use unique IDs/batch numbers instead of patient names for well identification. Record extraction plate layouts electronically and print them out to avoid manual transcription. Use barcodes for end-to-end scanning and linkage where feasible.<br>• **Review red flags:** Discrepancies between current and prior results (e.g., positive-to-negative or negative-to-positive conversions) are important warning signs. Reviewers should include "comparison with prior test history" in their review checklist.<br>• **System optimization:** The LIS should automatically display the patient's prior results side-by-side on the review screen and trigger alerts for significant changes or positive/negative conversion.

Field	Content
**Discussion Questions**	1. What risk factors in the extraction plate well-recording step contributed to this error? How should the laboratory standardize this procedure to prevent similar errors?<br>2. What are the advantages and disadvantages of manual recording versus electronic recording (e.g., ELN/LIS-integrated modules) in maintaining the identity chain? What improvements should be prioritized when resources are limited?<br>3. What clues led the reviewer to suspect a misassignment? How can these clues be standardized into "review checkpoints"?<br>4. Although the error was intercepted before release, what systemic issues does this case expose? How should the laboratory improve to prevent similar errors?
**Suggested Format**	Case discussion + recording workflow standardization exercise + review checklist design (30 minutes)

Category 4: Specimen Rejection Scenarios

Case C8: Clinical Microbiology — Blood Culture Contamination

Field	Content
**Case ID**	C8
**Rotation**	Clinical Microbiology
**Core Topic**	Blood culture collection standards and specimen rejection criteria
**Scenario**	A set of blood culture specimens (aerobic + anaerobic bottles) was submitted from a ward, with the requisition indicating "two-site, two-bottle collection" but only one set actually sent. After 48 hours, the aerobic bottle flagged positive with Gram-positive cocci on smear; the anaerobic bottle showed no growth. The clinician called the Microbiology laboratory requesting species identification and antimicrobial susceptibility testing (AST) results. However, the patient had no fever, chills, or other signs of infection; the collection site disinfection procedure was not documented; and the two draws were performed less than 30 minutes apart. The specimen was highly suspicious for skin flora contamination.
**Triple Helix Dimensions**	☑ Quality ☑ Safety ☑ Patient-Centered
**Learning Objectives**	1. Master blood culture collection standards and specimen rejection criteria;<br>2. Understand the impact of contaminated specimens on antimicrobial susceptibility testing (AST) result interpretation and antimicrobial stewardship.<br>3. Practice communication skills and scripts for conveying rejection decisions to clinicians.
**Teaching Points**	• Criteria for determining blood culture contamination: single-bottle positivity, organism type (e.g., coagulase-negative staphylococci), absence of clinical signs of infection, and nonstandard collection;<br>• Harms of false-positive/false-susceptibility results on antimicrobial use (antibiotic overuse, resistance induction, increased patient costs);<br>• Role of microbiology testing in antimicrobial stewardship (AMS)

Field	Content
	programs;<br>• Communication principles: reject the specimen, not the patient—approach as a clinical decision supporter rather than an "enforcer."
**Discussion Questions**	1. If the clinician insists on receiving a report despite your rejection, what is your justification and legal basis?<br>2. How would you recommend recollecting the specimen to the clinician without damaging the collaborative relationship?<br>3. If this specimen were accepted and a falsely susceptible AST result reported, what harm could the patient suffer?
**Suggested Format**	Case discussion + clinician-laboratory communication role-play (30 minutes)

Case C9: Outpatient/Emergency Lab — Incompletely Labeled Outpatient Specimen

Field	Content
**Case ID**	C9
**Rotation**	Outpatient/Emergency Laboratory
**Core Topic**	Outpatient specimen receipt and pre-phlebotomy patient education
**Scenario**	A 65-year-old female outpatient completed her requisition form and underwent phlebotomy at the collection window without informing the nurse that she was taking warfarin. The specimen tube label contained only the patient's name ("Fang Li") and age ("65")—no outpatient number, clinical diagnosis, or medication information. Upon arrival at the laboratory, the coagulation (PT/INR) result was markedly prolonged (INR 4.8). With no clinical information to assist interpretation, the verifying technologist faced a dilemma: releasing the result as-is might lead to inappropriate warfarin dosing, while withholding it would cause reporting delay.

Field	Content
**Triple Helix Dimensions**	☑ Quality ☑ Safety ☑ Patient-Centered
**Learning Objectives**	1. Recognize the importance of pre-phlebotomy patient information collection (medication history, diagnosis) for result interpretation;<br>2. Establish "minimum information standards" for outpatient specimen receipt;<br>3. Master management strategies for abnormal results with missing clinical information.
**Teaching Points**	• Information gaps and risks in the pre-analytical phase (pre-analytical errors account for 60%-70% of total laboratory errors);<br>• Key points for outpatient education: proactively disclose medication history (especially anticoagulants, antiplatelets, biotin), allergies, and fasting status;<br>• Three management options for incomplete specimens: reject and recollect, contact clinician for supplementary information, or release with "information incomplete" notation;<br>• Warfarin therapy INR monitoring range and bleeding risk.
**Discussion Questions**	1. How can patient education be efficiently delivered in a time-constrained phlebotomy window?<br>2. If the clinician cannot be reached, should the report be released? What annotations should be added if so?<br>3. What systemic issues does this case reflect in the outpatient workflow, and how could they be improved?
**Suggested Format**	Case discussion + patient education scenario simulation (25 minutes)

Category 5: Result Review Discrepancies/Reporting Errors

Case C10: Clinical Chemistry — Result-Clinical Diagnosis Mismatch

Field	Content
**Case ID**	C10
**Rotation**	Clinical Chemistry
**Core Topic**	Clinical information integration and anomaly identification in result verification
**Scenario**	A 58-year-old male patient's liver function results were: ALT 480 U/L, AST 520 U/L, GGT 320 U/L. The admission diagnosis was documented as "chronic gastritis," with no history of alcohol use or liver disease recorded in the medical record. The verifying technologist reviewed and released the report through the routine workflow. The next day, the clinician called questioning the results, stating they were "implausibly elevated" and suspecting specimen or reporting errors. Upon chart review, the patient had a long history of heavy alcohol consumption (approximately 250 mL of baijiu daily for 20 years), which he had not disclosed upon admission due to unrelated chief complaints. The actual diagnosis was alcoholic liver disease.
**Triple Helix Dimensions**	☑ Quality ☑ Safety ☑ Patient-Centered
**Learning Objectives**	1. Establish risk awareness to proactively investigate results that are inconsistent with clinical information;<br>2. Develop the ability to distinguish between "abnormal but plausible" vs. "abnormal and suspicious" results during verification;<br>3. Practice communication skills for confirming abnormal results with clinicians.
**Teaching Points**	• The "triangulation" principle in result verification: cross-validate result value + clinical history + prior results;<br>• Pattern recognition in liver enzyme abnormalities: hepatocellular pattern (ALT/AST markedly elevated) vs. cholestatic pattern (ALP/GGT markedly elevated);<br>• Management strategies when clinical information is missing: query

Field	Content
	the medical record, contact the clinical team, or hold the report;<br>• Mindset: "Abnormal does not necessarily mean unreasonable";<br>• Laboratory differentiation between alcoholic liver disease and viral hepatitis.
**Discussion Questions**	1. What "red flags" in this case should have alerted the verifying technologist?<br>2. If the patient's medical record contains no documented history of alcohol use, how would you handle this result?<br>3. Does the verifying technologist have a responsibility to proactively obtain clinical information? Where is the boundary?
**Suggested Format**	Case discussion + result verification simulation (20 minutes)

Case C11: Molecular Diagnostics — False-Positive Respiratory Pathogen Nucleic Acid Test Result Due to PCR Contamination

Field	Content
**Case ID**	C11
**Rotation**	Molecular Diagnostics / Clinical Microbiology
**Core Topic**	PCR contamination control and risk of false-positive results in respiratory pathogen testing
**Scenario**	A 4-year-old male with fever and cough for 3 days underwent respiratory pathogen multiplex PCR testing. The result was "Mycoplasma pneumoniae positive (Ct 36.5)." Azithromycin was prescribed. After 3 days of treatment, the patient's symptoms did not improve and chest X-ray showed progression. A repeat nucleic acid test and serology were both negative.<br><br>Retrospective review of the original run showed that the patient's sample (well E08) was adjacent to a strong positive control (E06, Ct 22.3) and another positive sample (E07, Ct 24.1). The batch positivity rate was 7/32 (>20%), far exceeding the seasonal baseline

Field	Content
	(5–8%). Ct values were widely dispersed (22–38). The positive control tube had been opened improperly, and aerosol cross-contamination was highly suspected. The initial report was confirmed as a false positive, resulting in unnecessary antibiotic use without clinical benefit.
**Triple Helix Dimensions**	☑ Quality ☑ Safety ☑ Patient-Centered
**Learning Objectives**	1. Understand common sources of PCR contamination in respiratory pathogen multiplex testing (positive controls, amplicons, specimen cross-contamination);<br>2. Master key contamination prevention strategies (physical partitioning, unidirectional workflow, UNG/dUTP system, proper pipetting sequence);<br>3.Identify laboratory clues (abnormally high positivity rates, Ct value dispersion, abnormal controls) and clinical clues (clinical inconsistency, treatment non-response) suggestive of false positives;<br>4.Master the approach to interpreting weak positive results (high Ct values) in conjunction with clinical and epidemiological data.
**Teaching Points**	**Contamination Sources**<br>• Aerosols from opening/pipetting positive controls<br>• Aerosols from opening post-amplification products (most common)<br>• Cross-contamination between specimens (splashing between adjacent wells, tip contamination)<br>• Environmental/consumable residues<br>**Clues Suggestive of Contamination**<br>• Positivity rate significantly above epidemiological baseline<br>• Dispersed Ct values (strong and weak positives coexisting)<br>• Positive signal in negative controls or aberrant Ct shift in positive controls<br>• Spatial clustering of positive samples on the extraction plate<br>**Prevention Strategies**<br>• Physical partitioning, unidirectional workflow, dedicated equipment<br>• UNG/dUTP carryover prevention system

Field	Content
	• Add specimens before positive controls; change tips between each addition<br>• Use low-concentration positive controls; handle in biosafety cabinet<br>• Regular decontamination of workbenches and pipettes<br>**Result Review**<br>• Compare batch positivity rate against baseline data<br>• Weak positives (Ct > 35) should be interpreted cautiously with clinical correlation<br>• Initiate contamination investigation when anomalies are detected (spatial distribution, operation records, control results)
**Discussion Questions**	1. What clues could the laboratory have identified before report release to detect and intercept the false-positive result in this case?<br>2. How can "abnormally elevated positivity rate" in respiratory pathogen multiplex testing be operationalized as a review indicator? What thresholds should trigger an alert?<br>3. How should a weak positive (Ct > 35) *Mycoplasma pneumoniae* result be reported? What comments should be included to guide appropriate clinical interpretation?<br>4. Following a contamination event, how should the laboratory conduct a thorough investigation? What steps should be taken to prevent recurrence?
**Suggested Format**	Case discussion + contamination investigation exercise + laboratory layout optimization exercise (35 minutes)

Category 6: Adverse Event/Near-Miss

Case C12: Clinical Immunology — Near-Miss Reporting Error

Field	Content
**Case ID**	C12
**Rotation**	Clinical Immunology
**Core Topic**	Near-miss identification, reporting, and systematic improvement
**Scenario**	A patient with suspected systemic lupus erythematosus (SLE) had a positive antinuclear antibody (ANA) result (titer 1:320, homogeneous pattern), with both anti-dsDNA and anti-Sm negative. The verifying technologist inadvertently clicked "anti-dsDNA positive" in the LIS (mouse selection error) and was about to click "review and release." At that moment, a senior technologist at the adjacent workstation noticed the screen and immediately intervened to stop the release. The error was corrected, and the report was never formally issued. The technologist involved believed that since "it was never released, it does not need to be reported" and planned not to document the event.
**Triple Helix Dimensions**	☑ Quality ☑ Safety ☑ Patient-Centered
**Learning Objectives**	1. Distinguish between adverse events and near-misses, and understand the reporting value of each;<br>2. Establish a "non-punitive reporting" patient safety culture and a "learning from errors" mindset;<br>3. Explore system-level interventions to prevent reporting errors.
**Teaching Points**	• Definition and distinction between adverse event and near-miss;<br>• Rationale for non-punitive reporting systems (errors stem from system deficiencies, not individual failures);<br>• Value of near-misses: they expose system vulnerabilities without patient harm—"free information" for quality improvement;<br>• LIS error-proofing design: dual-reviewer systems, mandatory confirmation dialogs, audit trail logs;<br>• Clinical weight of anti-dsDNA in SLE diagnosis (1982 ACR

Field	Content
	classification criteria);<br>• Clinical consequences of SLE misdiagnosis (inappropriate use of immunosuppressants).
**Discussion Questions**	1. Why should "it was never released" not be a justification for not reporting?<br>2. What technical measures could the LIS system implement to reduce the risk of mouse mis-clicks?<br>3. How can a "non-punitive reporting" culture be fostered within the laboratory to encourage voluntary near-miss reporting?
**Suggested Format**	Case discussion + safety culture debate (30 minutes)

4. Recommended Case Mapping by Rotation

Rotation	Primary Cases	Secondary Cases
Clinical Chemistry	C1, C5, C10	C4
Clinical Immunology	C3, C12	—
Clinical Microbiology	C8	—
Clinical Hematology/Body Fluids	C2, C6	—
Molecular Diagnostics	C7, C11	—
Outpatient/Emergency Lab	C4, C9	—

Note: The mapping above represents primary recommendations; preceptors may flexibly select other cases based on rotation-specific teaching contexts.

5. Instructions for Preceptors

Timing for use:

- Morning reports
- Shift handovers
- Designated teaching sessions
- Real-time workflow pauses

Recommended session duration:
20–40 minutes per case, adjustable based on case complexity and student engagement.

Recommended discussion flow:

Step	Activity	Duration
1	Preceptor introduces the case scenario (facts only, without conclusions)	2 min
2	Students independently analyze and formulate initial judgments	3 min

Step	Activity	Duration
3	Small-group discussion (3–5 students per group, addressing discussion questions)	10–15 min
4	Group reporting + preceptor summary and content reinforcement	10–15 min

Teaching philosophy:

- The goal of case discussion is not to arrive at "the correct answer," but to **reveal blind spots, exercise clinical reasoning, and reinforce safety awareness**.
- Preceptors should encourage diverse perspectives and foster an open, non-punitive discussion environment.
- Students should be encouraged to connect cases to their own rotation experiences, facilitating transfer from cases to practice.

Supplementary Material S2:

Quality-Safety-Patient-Centered" Triple Helix Teaching Model — Scenario-Based Simulation Training Scenarios and Assessment Tools

1 Instructions for Use

1.1 Purpose and Scope

This material serves as a supporting training tool for the "Quality-Safety-Patient-Centered" Triple Helix laboratory internship teaching model. It focuses on high-frequency scenarios in laboratory medicine internships involving communication conflicts, safety risks, and priority management. The aim is to translate quality control, safety governance, and patient service concepts into actionable clinical situational training.

Target Audience: Undergraduate/college clinical interns in Medical Laboratory Science

Applicable Scenarios: Scenario-based simulation teaching, formative assessment, and comprehensive departmental exit evaluations at various internship stages

Corresponding Teaching Objectives: To strengthen interns' whole-process quality awareness, systematic safety literacy, and patient-centered service capabilities, addressing the deficiencies of traditional teaching which "emphasizes operations, neglects communication, and lacks systems thinking"

1.2 Standardized Implementation Process

1.2.1 **Preparation:** Instructors familiarize themselves with scenario scripts and assessment checklists in advance, arrange 1–2 assistants to play clinical staff/patient roles, and prepare corresponding props (specimen tubes, telephones, test requisition forms, etc.).

1.2.2 **Scenario Exercise:** Interns independently complete scenario tasks, with each scenario lasting 5–10 minutes. Instructors observe throughout and simultaneously record notes using the assessment checklist.

1.2.3 **Feedback and Debriefing:** After the exercise, instructors provide targeted feedback based on the assessment checklist, analyzing behavioral strengths and areas for improvement across the three dimensions of "quality bottom line, safety red line, and patient-centeredness."

1.2.4 **Score Entry:** Individual scenario scores are entered into the comprehensive scoring summary table and incorporated into the intern's Triple Helix comprehensive competency evaluation system.

1.3 Scoring Rules

The Mini-CEX universal 9-point scoring scale is adopted:

- **1–3 points (Unsatisfactory):** Fails to meet basic job requirements, with significant safety/quality risks.
- **4–6 points (Satisfactory):** Basically, meets standards, with no errors in core processes; communication and service skills require improvement.
- **7–9 points (Excellent):** Processes are complete and standardized; communication is efficient and appropriate; risk prediction and problem-solving abilities are outstanding.

Each scenario includes **5 core assessment dimensions** of equal weight. The composite score is the arithmetic mean of all dimension scores. **All scenarios use the same five dimensions**, in the following fixed order: Professionalism, Communication Skills, Quality Awareness, Safety Behavior, Patient Service.

1.4 Correspondence with the Core Evaluation System

This assessment tool aligns with the study's core evaluation indicators as follows:

- **Quality Awareness** and **Safety Behavior** dimensions correspond to "Process Quality and Safety Indicators"
- **Communication Skills** and **Patient Service** dimensions correspond to "Patient Satisfaction and Clinical Department Satisfaction"
- **Professionalism** dimension corresponds to the value internalization aspect of the "Triple Helix Comprehensive Competency Scale"
- The overall score can serve as an important component of the Mini-CEX comprehensive evaluation

2 Overview of Scenario-Based Simulation Training Scenarios

Table S2: Basic Information Summary of 8 Scenario-Based Simulation Scenarios

Scenario No.	Scenario Title	Corresponding Triple Helix Core Dimensions	Difficulty Level	Applicable Internship Stage

1	Critical Value Notification for Hyperkalemia in General Ward (Physician Challenges Results)	Safety Management, Quality Compliance, Patient-Centeredness	Intermediate	Mid-internship
2	Critical Value Notification for Low Platelets in Pediatric Emergency (Anxious Family Member + Absent Designee)	Patient-Centeredness, Safety Management, Communication & Collaboration	Intermediate-Advanced	Mid-to-late internship
3	Rejection Communication for Routine Coagulation Specimen (Clotted Sample + Clinician Requests Compromised Testing)	Quality Compliance, Communication & Collaboration, Patient-Centeredness	Intermediate	Mid-internship
4	Rejection Communication for Emergency Preoperative Specimen (Insufficient Volume + Surgeon Pressure)	Safety Management, Quality Compliance, Emergency Decision-Making	Intermediate-Advanced	Mid-to-late internship

5	Identity Verification for Same-Name Patients at Outpatient Phlebotomy Window	Safety Management, Patient-Centeredness, Quality Compliance	Basic	Early internship
6	Traceability Verification for Same-Name Patient Specimens at Specimen Receiving Station	Safety Management, Quality Compliance, Problem-Solving	Intermediate-Advanced	Mid-internship
7	Prioritization of Multiple Parallel Emergency Specimens	Quality-Efficiency Balance, Safety Management, Clinical Collaboration	Intermediate-Advanced	Mid-to-late internship
8	STAT Specimen Prioritization During CPR Emergency	Patient Safety, Emergency Management, Cross-Functional Collaboration	Advanced	Late internship

Module 1: Critical Value Telephone Notification Scenarios

Scenario 1: Critical Value Notification for Hyperkalemia in General Ward (Physician Challenges Results)

Corresponding Dimensions: Safety Management, Quality Compliance, Patient-Centeredness
Difficulty Level: Intermediate
Applicable Stage: Mid-internship
Setting Context: Clinical Chemistry laboratory *(adaptable to other disciplines for critical value communication training)*

Context

An intern, while reviewing reports in the biochemistry laboratory, identifies a serum potassium result of 6.8 mmol/L (reference range: 3.5–5.5 mmol/L) for a hospitalized patient in the gastroenterology department, meeting the critical value reporting criteria. At this time, the ward is in the peak morning rounds period. The attending physician answers the phone in a hurried tone, questions the accuracy of the result, and requests to simplify the reporting process, refusing to cooperate with the complete critical value read-back and registration procedure.

Role Assignment

- **Intern:** Laboratory technologist responsible for critical value reporting and closed-loop management
- **Simulated Role:** Attending physician (impatient, questions results, refuses to cooperate with registration)
- **Evaluator:** Instructor

Core Training Objectives

1. Standardize the implementation of the complete critical value reporting process to ensure accurate and traceable information transfer
2. Respond professionally to clinical questions about results, demonstrating quality management reasoning
3. Maintain safety standards while preserving good collaborative relationships under clinical pressure

Execution Flow

1. **Trigger:** Intern identifies critical value, completes specimen re-testing for confirmation, and calls the ward
2. **Conflict:** Physician claims to be busy, questions the result, and asks for direct verbal reporting
3. **Decision:** Balance communication efficiency with process compliance, respond to questions, and emphasize the clinical risks of the critical value
4. **Closure:** Confirm recipient identity, complete result read-back, fully document reporting information, and simultaneously alert clinical staff to key management points

Table S2-1: Assessment Checklist for Scenario 1

**Assessment Dimension**	**Observation Points**	**Score (1–9)**
Quality Awareness	1. Completes specimen re-testing before reporting to confirm result accuracy<br>2. Accurately reports patient basic information, test item, result, and units	
Safety Behavior	1. Verifies recipient name and employee ID, performs result read-back confirmation<br>2. Completely documents reporting time, recipient, and content<br>3. Proactively highlights the core clinical risks of this critical value	
Communication Skills	1. Maintains professional and calm tone, does not simplify core processes due to clinical urgency<br>2. Provides evidence-based responses to result challenges (citing internal quality control status, re-test consistency)<br>3. Demonstrates empathy, conveys key information concisely	
Patient Service	1. Proactively informs clinical staff of the official report issuance time<br>2. Frames communication around patient safety rather than merely emphasizing procedural compliance	

Professionalism	Adheres fully to laboratory standards, upholds quality and safety bottom lines, does not make arbitrary concessions	
Composite Score	——	

Scenario 2: Critical Value Notification for Low Platelets in Pediatric Emergency (Anxious Family Member + Absent Designee)

Corresponding Dimensions: Patient-Centeredness, Safety Management, Communication & Collaboration
Difficulty Level: Intermediate-Advanced
Applicable Stage: Mid-to-late internship
Setting Context: Emergency/Outpatient Laboratory

Context

During the night shift in the emergency laboratory, a complete blood count result for a 3-year-old febrile child shows a platelet count of 18×10^9/L, meeting critical value criteria. The intern calls the emergency nurses' station, and the nurse responds that all physicians are busy in the resuscitation room and can only relay the message. Simultaneously, the child's family member approaches the window to inquire about the results, visibly anxious, repeatedly asking about the diagnosis.

Role Assignment

- **Intern:** Emergency laboratory duty staff
- **Simulated Role 1:** Emergency nurse (busy, not qualified to receive critical values)
- **Simulated Role 2:** Child's family member (anxious, repeatedly asking about the condition)
- **Evaluator:** Instructor

Core Training Objectives

1. Ensure critical values are received by a qualified physician to complete the compliant closed loop
2. Respond appropriately to family inquiries while maintaining professional boundaries
3. Maintain order under dual pressure, balancing medical safety with patient experience

Execution Flow

1. **Trigger:** Identifies critical platelet value, confirms via re-testing, and attempts to report
2. **Dual Conflict:** Nurse is not qualified to receive; family member crowds the window with questions
3. **Decision:** Prioritize contacting the attending physician on duty to complete reporting, while simultaneously calming the family at the window

4. **Closure:** Complete critical value registration loop, guide family to the consultation room to wait for physician explanation

Table S2-2: Assessment Checklist for Scenario 2

Assessment Dimension	Observation Points	Score (1–9)
Quality Awareness	Completes specimen re-testing and smear confirmation before reporting critical value, rules out interfering factors such as clots	
Safety Behavior	1. Insists that critical values be received by a licensed physician, does not treat nurse relay as closure<br>2. Does not interpret diagnostic conclusions to family members, avoids overstepping and causing medical risks<br>3. Completely documents the full reporting process	
Communication Skills	1. Comforts family members with a gentle tone, uses plain language to explain "the result is abnormal and the doctor has been notified immediately"<br>2. Remains composed under pressure, clearly explains procedures and wait times<br>3. Communicates with healthcare staff efficiently and accurately without information omission	
Patient Service	1. Empathizes with family anxiety, does not dismiss them brusquely<br>2. Proactively informs of report issuance time and report pickup location<br>3. Guides family to the consultation room to wait, avoiding crowding at the window	
Professionalism	Maintains professional boundaries while demonstrating humanistic care	
Composite Score	——	

Module 2: Specimen Rejection Communication Scenarios

Scenario 3: Rejection Communication for Routine Coagulation Specimen (Clotted Sample + Clinician Requests Compromised Testing)

Corresponding Dimensions: Quality Compliance, Communication & Collaboration, Patient-Centeredness
Difficulty Level: Intermediate
Applicable Stage: Mid-internship

Setting Context: Specimen Reception *(adaptable to hematology for body fluid specimen rejection training)*

Context

A nurse from the neurology department delivers a coagulation specimen. The intern examines it and finds small clots within the specimen, rendering it non-compliant with testing quality requirements and subject to rejection per protocol. The nurse states that the patient has difficult venous access, pleads to run the specimen anyway, and claims to assume responsibility for the consequences.

Role Assignment

- **Intern:** Specimen receiving station staff
- **Simulated Role:** Neurology nurse (reluctant, emphasizes collection difficulty, requests compromised testing)
- **Evaluator:** Instructor

Core Training Objectives

1. Clearly articulate the rejection reason based on specimen quality standards, upholding quality bottom lines
2. Persuade the other party using clinical risk logic to avoid escalation
3. Provide feasible optimization recommendations to demonstrate collaborative spirit

Execution Flow

1. **Trigger:** Discovers clots during specimen inspection, informs nurse of rejection
2. **Conflict:** Nurse emphasizes collection difficulty and questions rejection criteria
3. **Decision:** Explains the impact of clots on coagulation results and clinical medication risks, provides collection optimization suggestions
4. **Closure:** Completes rejection documentation properly, simultaneously communicates re-collection precautions

Table S2-3: Assessment Checklist for Scenario 3

**Assessment Dimension**	**Observation Points**	**Score (1–9)**
Quality Awareness	1. Accurately identifies the reason for specimen rejection and its impact on test results<br>2. Strictly enforces rejection criteria, does not relax quality requirements arbitrarily<br>3. Completes rejection documentation properly, retains complete records	
Safety Behavior	Proactively explains the potential clinical medication risks (e.g., misinterpretation of anticoagulant dosage) that substandard specimens may cause	
Communication Skills	1. First acknowledges collection difficulty, then explains rejection rationale<br>2. Uses clinical risk language rather than rigid "policy" statements<br>3. Responds to questions with evidence-based reasoning, avoids escalating conflict	
Patient Service	1. Provides optimization suggestions for re-collection procedures<br>2. Promises priority testing for the re-submitted specimen to reduce patient waiting time	
Professionalism	Upholds quality standards while demonstrating collaboration, avoids blame-shifting	
Composite Score	——	

Scenario 4: Rejection Communication for Emergency Preoperative Specimen (Insufficient Volume + Surgeon Pressure)

Corresponding Dimensions: Safety Management, Quality Compliance, Emergency Decision-Making
Difficulty Level: Intermediate-Advanced
Applicable Stage: Mid-to-late internship

Setting Context: Emergency/Outpatient Laboratory

Context

An emergency appendectomy is about to begin. The operating room delivers the patient's preoperative infection screening specimen, but the collected blood volume is only half of the required amount, failing to meet testing requirements. The surgeon calls to apply pressure, stating that the patient has already been anesthetized, requesting that the test be performed anyway and claiming to assume full responsibility.

Role Assignment

- **Intern:** Emergency laboratory duty staff
- **Simulated Role:** Surgeon (firm tone, emphasizes surgical urgency)
- **Evaluator:** Instructor

Core Training Objectives

1. Assess specimen quality risks in emergency scenarios while maintaining safety standards
2. Engage in shared decision-making with clinical staff and propose feasible alternatives
3. Document communication records properly and clarify accountability boundaries

Execution Flow

1. **Trigger:** Identifies insufficient specimen volume during inspection, informs that re-collection is required
2. **Conflict:** Surgeon calls to pressure, emphasizing surgical urgency
3. **Decision:** Explains testing risks of insufficient volume, proposes an alternative plan (dilution testing with annotation) and clearly states its limitations
4. **Closure:** If the clinician insists on compromised testing, retain communication records and annotate specimen abnormality on the report

Table S2-4: Assessment Checklist for Scenario 4

Assessment Dimension	Observation Points	Score (1–9)

Quality Awareness	1. Accurately explains the impact of insufficient specimen volume on testing methodology<br>2. Clearly annotates specimen abnormality status on the report if compromised testing is performed<br>3. Completely retains communication records and clinical opinions	
Safety Behavior	1. Fully informs the physician of the uncertainty and clinical risks of compromised test results<br>2. Does not arbitrarily agree to unsupported compromised testing; escalates to instructor when necessary	
Communication Skills	1. First acknowledges surgical urgency, then clearly explains risks—does not adopt an adversarial stance<br>2. Proactively offers alternatives rather than simply rejecting the request<br>3. Communicates efficiently without delaying emergency surgery	
Patient Service	Centers the discussion around patient perioperative safety	
Professionalism	Maintains composure under emergency conditions, clarifies accountability, does not improperly promise result accuracy	
Composite Score	——	

Module 3: Same-Name Patient Identity Verification Scenarios

Scenario 5: Identity Verification for Same-Name Patients at Outpatient Phlebotomy Window

Corresponding Dimensions: Safety Management, Patient-Centeredness, Quality Compliance
Difficulty Level: Basic
Applicable Stage: Early internship

Setting Context: Outpatient Phlebotomy

Context

At the outpatient phlebotomy window, two patients with identical names present their barcode labels one after another, corresponding to different departments and test panels. One of the patients is rushing to have blood drawn. Relying solely on name verification would easily lead to specimen misidentification.

Role Assignment

- **Intern:** Outpatient phlebotomy technician
- **Simulated Role 1:** Impatient patient (rushing the phlebotomy)
- **Simulated Role 2:** Regular patient (waiting calmly)
- **Evaluator:** Instructor

Core Training Objectives

1. Strictly implement multi-dimensional patient identification protocols to prevent specimen mix-ups among same-name patients
2. Complete verification efficiently while maintaining a positive patient experience
3. Explain the purpose of verification to patients to enhance cooperation

Execution Flow

1. **Trigger:** Two same-name patients arrive at the window simultaneously, one of them rushing
2. **Risk Point:** Name-only verification would lead to specimen misidentification
3. **Decision:** Apply triple verification—"Name + Medical Record Number/ID Number + Test Panel"—confirming each patient sequentially
4. **Closure:** Re-verify tube barcodes against patient information after phlebotomy

Table S2-5: Assessment Checklist for Scenario 5

**Assessment Dimension**	**Observation Points**	Score (1–9)
Quality Awareness	Performs two-way verification of specimens and patient information both before and after phlebotomy	
Safety Behavior	1. Does not rely solely on name; simultaneously verifies unique identifiers and test panels<br>2. Keeps specimens from the two patients physically separated to avoid confusion<br>3. Rechecks information after labeling tubes	
Communication Skills	1. Maintains complete verification process despite patient rush, with a gentle tone<br>2. Proactively explains the purpose of multi-dimensional verification to gain patient understanding	
Patient Service	Balances efficiency, avoids excessive patient wait times, and demonstrates respect during verification	
Professionalism	Strictly follows patient identification protocols (verifying patient name, medical record number, date of birth, test panel, and specimen label consistency both before and after collection), does not cut corners due to patient pressure.	
Composite Score	——	

Scenario 6: Traceability Verification for Same-Name Patient Specimens at Specimen Receiving Station

Corresponding Dimensions: Safety Management, Quality Compliance, Problem-Solving
Difficulty Level: Intermediate-Advanced
Applicable Stage: Mid-internship

Setting Context: Specimen Reception *(adaptable to molecular diagnostics for nucleic acid extraction traceability training)*

Context

A batch of inpatient specimens arrives. Among them, two specimens have blurry barcodes with only the same name legible. They come from two different departments and have overlapping test panels, making direct matching to patient information impossible.

Role Assignment

- **Intern:** Specimen receiving station staff
- **Simulated Role:** Ward nurse (on the phone, impatient with the verification process)
- **Evaluator:** Instructor

Core Training Objectives

1. Accurately match specimen ownership through multi-dimensional information tracing
2. Properly handle poorly labeled specimens to prevent misattribution of reports
3. Provide feedback on front-end issues to drive process improvement

Execution Flow

1. **Trigger:** Identifies two specimens with same name and blurry barcodes during inspection
2. **Investigation:** Contacts the corresponding departments to cross-verify bed numbers, hospital registration numbers, collection times, and other information
3. **Decision:** If information cannot be fully confirmed, rejects the specimens as non-compliant rather than risking testing
4. **Closure:** Documents problematic specimens and provides feedback to clinical staff on barcode standards

Table S2-6: Assessment Checklist for Scenario 6

Assessment Dimension	Observation Points	Score (1–9)

Quality Awareness	1. Does not guess specimen ownership arbitrarily; only signs off after confirmation<br>2. Properly documents problematic specimens and retains traceability records	
Safety Behavior	1. Cross-verifies patient information using multiple dimensions<br>2. Firmly rejects specimens when information cannot be verified, preventing report misattribution<br>3. Simultaneously provides feedback to clinical front-end to drive continuous improvement	
Communication Skills	1. Communicates clearly and logically with the ward, specifying verification requirements<br>2. Explains safety risks to clinical staff when they are impatient, gaining their cooperation	
Patient Service	Maintains focus on patient safety as the ultimate goal of traceability verification	
Professionalism	1. Upholds patient safety standards, does not relax criteria due to heavy workload<br>2. Maintains professional composure when communicating with ward staff	
Composite Score	——	

Module 4: Emergency Specimen Prioritization Scenarios

Scenario 7: Prioritization of Multiple Parallel Emergency Specimens

Corresponding Dimensions: Quality-Efficiency Balance, Safety Management, Clinical Collaboration
Difficulty Level: Intermediate-Advanced
Applicable Stage: Mid-to-late internship

Setting Context: Emergency/Outpatient Laboratory

Context

During the morning peak period, the emergency laboratory simultaneously receives three types of specimens: a chest pain center STEMI specimen (requiring result reporting within 20 minutes of specimen receipt), a stroke center intracranial hemorrhage specimen (requiring result reporting within 30 minutes of specimen receipt), and a routine emergency abdominal pain specimen (requiring result reporting within 60 minutes of specimen receipt). Only one biochemistry analyzer channel is available. The routine emergency nurse repeatedly calls to urge results.

Role Assignment

- **Intern:** Emergency biochemistry duty staff
- **Simulated Role:** Routine emergency nurse (calling to urge results)
- **Evaluator:** Instructor

Core Training Objectives

1. Establish reasonable testing priorities in accordance with green channel protocols
2. Effectively address clinical requests and manage expectations
3. Ensure prioritization does not compromise testing quality

Execution Flow

1. **Trigger:** Three types of specimens arrive simultaneously; instrument resources are insufficient
2. **Conflict:** Routine emergency nurse urges and questions the testing order
3. **Decision:** Prioritize according to critical illness severity, explain the rationale and estimated time to the routine emergency nurse
4. **Closure:** Complete green channel specimens first and push results, maintaining quality control throughout

Table S2-7: Assessment Checklist for Scenario 7

Assessment Dimension	Observation Points	Score (1–9)
Quality Awareness	1. Prioritization does not affect internal quality control execution or operational standards<br>2. STAT specimens still undergo result review criteria; reports are not issued carelessly	
Safety Behavior	1. Strictly follows emergency green channel priority protocols<br>2. Clear labeling of specimens by priority level to avoid confusion and mis-issuance	
Communication Skills	1. Explains prioritization rules with evidence-based reasoning, demonstrating professional standards<br>2. Proactively communicates estimated wait times to address clinical concerns<br>3. Effectively coordinates with clinical staff regarding the priority arrangement	
Patient Service	Minimizes wait times for routine specimens while ensuring critical care prioritization	
Professionalism	1. Demonstrates orderly scheduling and clear processes under resource constraints<br>2. Remains calm and professional when responding to clinical urgings	
Composite Score	——	

Scenario 8: STAT Specimen Prioritization During CPR Emergency

Corresponding Dimensions: Patient Safety, Emergency Management, Cross-Functional Collaboration
Difficulty Level: Advanced
Applicable Stage: Late internship

Setting Context: Emergency/Outpatient Laboratory

Context

An intern is running a batch of routine outpatient biochemistry specimens when the resuscitation room delivers STAT specimens from a patient undergoing CPR, requesting immediate testing to guide medication. Interrupting the current run would delay all routine specimens; not prioritizing the STAT specimen could delay resuscitation efforts. The resuscitation room nurse repeatedly comes to the window to urge.

Role Assignment

- **Intern:** Biochemistry testing duty staff
- **Simulated Role:** Resuscitation room nurse (anxious, repeatedly urging)
- **Evaluator:** Instructor

Core Training Objectives

1. Make quick decisions to prioritize critical care specimens
2. Rationally allocate resources to minimize impact on routine testing
3. Maintain operational standards under emergency conditions while addressing multiple stakeholders' needs

Execution Flow

1. **Trigger:** STAT specimen from resuscitation room arrives; routine specimens are already loaded
2. **Decision:** Assess instrument capabilities for STAT insertion; activate backup rapid channel if necessary
3. **Response:** Respond to urgings with clear estimated time to results
4. **Closure:** Provide immediate verbal result reporting, followed by formal written report; explain delays to affected routine patients

Table S2-8: Assessment Checklist for Scenario 8

Assessment Dimension	Observation Points	Score (1–9)

Quality Awareness	STAT specimen testing still follows operational standards; results are reviewed and confirmed before release	
Safety Behavior	1. Prioritizes critical care specimens to ensure resuscitation timeliness<br>2. Accurately verifies specimen information, avoids errors due to haste<br>3. Verbal reports are followed by written reports, forming a complete loop	
Communication Skills	1. To resuscitation staff: provides timely progress updates with clear timelines to manage expectations<br>2. To affected patients: proactively explains the reason and offers apologies	
Patient Service	Balances the needs of critically ill patients with those of routine patients, ensuring that all receive appropriate care	
Professionalism	1. Remains calm under pressure, maintains orderly operations, balances efficiency and safety<br>2. Quickly assesses instrument scheduling options without hesitation or delay; actively calls for colleague support when necessary	
Composite Score	——	

4 Comprehensive Scoring Summary Tool

Table S2-9: Scenario-Based Simulation Training Comprehensive Scoring Summary

Intern Name		Internship Batch		Instructor			
Scenario No.	Scenario Title	Dimension Scores (1–9)					Composite Score
		Professionalism	Communication Skills	Quality Awareness	Safety Behavior	Patient Service	
1	Hyperkalemia Critical Value Notification						
2	Pediatric Platelet Critical Value Notification						
3	Coagulation Specimen Rejection Communication						
4	Preoperative Specimen Rejection Communication						
5	Same-Name Patient Phlebotomy Verification						
6	Same-Name Specimen Traceability Verification						
7	Emergency Specimen Prioritization						
8	STAT Specimen Prioritization						

	During CPR						
**Dimension Average Score**							
**Overall Evaluation**							
**Improvement Recommendations**							

Note: The scores from this summary table can be directly incorporated into the intern's Triple Helix comprehensive competency evaluation system as core data sources for formative assessment. The dimension average scores show high correlation with Mini-CEX composite scores and Triple Helix Scale scores, serving as complementary validation for each other.

Instructions for completing this summary:

- **Dimension Average Score:** Calculate the arithmetic mean of each dimension across all 8 scenarios.
- **Overall Evaluation:** Provide a 1–2 sentence summary of the intern's overall performance across all scenarios.
- **Improvement Recommendations:** List 2–3 specific actionable suggestions for the intern's future development.

Supplementary Material S3:

Scale 1: Reflective Journal Scoring Rubric (5-point Likert Scale)

Dimension	Scoring Criteria	1 (Very Poor)	2 (Poor)	3 (Average)	4 (Good)	5 (Excellent)
1. Problem Identification	Ability to accurately identify key clinical/quality/safety issues	Failed to identify any key issues	Identified only surface-level issues, missing the core	Identified main issues but not comprehensively	Accurately identified all key issues	Precisely identified core issues and anticipated potential related issues
2. Analysis Depth	Ability to conduct in-depth root cause analysis from multiple dimensions (e.g., systems, processes, personnel, protocols)	Merely described phenomena without analysis	Analysis remained superficial, lacking evidence	Some analysis provided, but limited to a single dimension	Relatively in-depth analysis involving multi-dimensional causal relationships	Thorough and insightful analysis revealing systemic root causes
3. Solution Feasibility	Whether proposed improvement solutions are specific, actionable, and practical	No solution proposed or completely impractical	Solution overly idealistic, lacking operational feasibility	Solution generally feasible but lacking in detail	Solution specific and feasible, with consideration of implementation conditions	Highly feasible solution with clear step-by-step planning and good generalizability
4. Patient-Centered Awareness	Whether the reflection demonstrates patient-centered thinking, with attention to patient safety, experience, and needs	Completely ignored patient perspective	Occasionally mentioned patients but only superficially	Considered basic patient needs and safety	Paid full attention to patient experience, demonstrating empathy and safety awareness	Patient-centered perspective consistently maintained throughout, integrating safety with humanistic care

5. Self-Reflection	Ability to objectively evaluate personal limitations and identify future improvement directions	No self-reflection content included	Only vague self-evaluation without specific improvements	Able to identify limitations but improvement directions remain general	In-depth reflection with clear improvement goals	Deep reflection with specific, measurable improvement plans demonstrating growth mindset

Scoring Guide: 1 = Very Poor; 2 = Poor; 3 = Average; 4 = Good; 5 = Excellent. Total score ranges from 5 to 25.

Supplementary Material S4:

Scale 2: Case Tracking Report Scoring Rubric (Standardized Rubric)

Assessment Domain	Weight (%)	Scoring Criteria	Score (0–100)
**1. Case Description Completeness**	15	Whether the report completely describes patient demographics, laboratory findings, clinical context, and key timelines	/100
**2. Problem Analysis & Logical Reasoning**	25	Whether laboratory medicine knowledge is systematically applied to analyze the problem, with clear and evidence-supported logical reasoning	/100
**3. Interventions & Process Management**	20	Whether the actions taken, communication and coordination, and quality control processes are accurately described	/100
**4. Patient Safety & Quality Improvement Awareness**	20	Whether safety risks are identified and reasonable quality improvement recommendations are proposed	/100
**5. Report Structure & Academic Standard**	10	Whether the report is well-structured (background, process, analysis, conclusion) with professional and standardized language	/100
**6. Reflection & Transferability**	10	Whether lessons learned are summarized and insights with practical value for future practice are presented	/100

Total Score (Weighted Sum): ____ / 100

Grading Standards: Excellent: ≥90; Good: 80–89; Pass: 70–79; Needs Improvement: 60–69; Fail: <60

Note: S3 (Reflective Journal) is a process-oriented formative assessment of ongoing reflection across rotations; S4 (Case Tracking Report) is a summative assessment of integrated case analysis competence. Final score = 40% × (mean journal score) + 60% × (case report score).

Supplementary Material S5:

Observation Checklist for Implementation Fidelity of Triple Helix Teaching Sessions

Item	Yes	No	Partial	Notes
**A. Session Structure**				
Session started and ended on time as scheduled	□	□	□	
Standardized case bank or simulation scenarios were used	□	□	□	
Session duration met the planned requirement (≥90% of target)	□	□	□	
**B. Content Coverage – Quality Dimension**				
Total process quality control (pre-/analytical/post-analytical phases) was addressed	□	□	□	
IQC/Westgard rules or out-of-control management was discussed	□	□	□	
Result review logic and verification processes were emphasized	□	□	□	
**C. Content Coverage – Safety Dimension**				
Patient safety elements (identification/closed-loop critical value reporting/handoff/adverse events) were addressed	□	□	□	
Clinical consequences of safety lapses were discussed	□	□	□	
Closed-loop communication protocols were emphasized	□	□	□	
**D. Content Coverage – Patient-Centered Dimension**				

Impact of laboratory findings on clinical decision-making was addressed	☐	☐	☐	
Patient communication/education/special population care was emphasized	☐	☐	☐	
Interdisciplinary collaboration and humanistic care were demonstrated	☐	☐	☐	
**E. Teaching Methods**				
Students were encouraged to actively analyze rather than passively listen	☐	☐	☐	
Guided questioning was used to promote critical thinking	☐	☐	☐	
Immediate feedback was provided during the session	☐	☐	☐	
**F. Student Engagement**				
Most students actively participated in discussions or activities	☐	☐	☐	
Students had opportunities to ask questions and express opinions	☐	☐	☐	

Overall Fidelity Score: ____ / 100 (0 = not implemented as designed; 100 = fully implemented as designed)

Overall Rating:

☐ Excellent (≥90) – Fully delivered with all core elements covered ☐ Good (80–89) – Largely delivered with minor omissions

☐ Fair (70–79) – Partially delivered with multiple omissions ☐ Needs Improvement (<70) – Substantial deviation from protocol

Observer Signature: ________ Date: ________

Scoring: Yes = 1, Partial = 0.5, No = 0. Overall score (%) = (sum / total items) × 100. "Yes" = explicitly and adequately addressed; "Partial" = mentioned but insufficient depth or subset coverage; "No" = not addressed. N/A items excluded from calculation. Rate based on the session as a whole.

Supplementary Material S6:

Teaching Session Attendance and Completion Log

Instruction: Complete one row for each teaching session delivered.

Date	Rotation	Preceptor	Session Theme	Completion Status	If Not Completed as Planned, Specify Reason	Students Attending/Enrolled	Preceptor Signature
	clinical hematology and body fluid analysis		☐ Quality case discussion<br>☐ Safety simulation drill<br>☐ Patient-centered teaching	☐ Fully completed<br>☐ Partially completed<br>☐ Not completed<br>☐ Completed with modification		/	
	Clinical Chemistry		☐ Quality case discussion<br>☐ Safety simulation drill<br>☐ Patient-centered teaching	☐ Fully completed<br>☐ Partially completed<br>☐ Not completed<br>☐ Completed with modification		/	

	Clinical Immunology		☐ Quality case discussion<br>☐ Safety simulation drill<br>☐ Patient-centered teaching	☐ Fully completed<br>☐ Partially completed<br>☐ Not completed<br>☐ Completed with modification		/	
	Clinical Microbiology		☐ Quality case discussion<br>☐ Safety simulation drill<br>☐ Patient-centered teaching	☐ Fully completed<br>☐ Partially completed<br>☐ Not completed<br>☐ Completed with modification		/	
	Clinical Molecular Diagnostics		☐ Quality case discussion<br>☐ Safety simulation drill<br>☐ Patient-centered teaching	☐ Fully completed<br>☐ Partially completed<br>☐ Not completed<br>☐ Completed with modification		/	
	Outpatient/ Emergency Laboratory		☐ Quality case discussion<br>☐ Safety simulation drill<br>☐ Patient-centered teaching	☐ Fully completed<br>☐ Partially completed<br>☐ Not completed<br>☐ Completed with modification		/	

Supplementary Material S7:

Triple Helix Integrated Literacy Scale (THILS)

Instructions: Please indicate your level of agreement with each of the following statements regarding your clinical internship experience. For each item, circle the number that best reflects your opinion using the scale below.

**Response**	**Score**
Strongly Disagree	1
Disagree	2
Neutral	3
Agree	4
Strongly Agree	5

Dimension 1: Quality Literacy (12 items)

No.	Item	1	2	3	4	5
QL1	I am able to correctly interpret laboratory quality control charts and identify out-of-control signals.	1	2	3	4	5
QL2	I understand the principles and procedures for implementing corrective actions when quality control fails.	1	2	3	4	5
QL3	I consistently follow standard operating procedures (SOPs) for all laboratory tests I perform.	1	2	3	4	5
QL4	I am familiar with the ISO 15189 quality management system requirements for medical laboratories.	1	2	3	4	5
QL5	I can accurately document and report quality control results in accordance with departmental standards.	1	2	3	4	5
QL6	I understand the importance of regular equipment maintenance and calibration for ensuring test accuracy.	1	2	3	4	5
QL7	I am able to identify sources of pre-analytical, analytical, and post-analytical errors in laboratory testing.	1	2	3	4	5
QL8	I actively participate in quality improvement initiatives within my clinical rotation.	1	2	3	4	5
QL9	I understand the criteria for laboratory report verification and release and can ensure that reports meet quality standards.	1	2	3	4	5
QL10	I routinely review and verify test results for clinical plausibility before releasing them.	1	2	3	4	5
QL11	I am knowledgeable about the procedures for handling and documenting non-conforming test events.	1	2	3	4	5
QL12	I understand the role of internal quality control and external quality assessment in ensuring laboratory quality.	1	2	3	4	5

Dimension 2: Safety Literacy (12 items)

No.	Item	1	2	3	4	5
SL1	I consistently adhere to standard precautions when handling blood and other body fluid specimens.	1	2	3	4	5
SL2	I am able to correctly identify and respond to laboratory safety hazards (e.g., chemical spills, sharps injuries).	1	2	3	4	5
SL3	I consistently verify patient identity using at least two patient identifiers before specimen collection or testing.	1	2	3	4	5
SL4	I understand the proper procedures for safe handling, transportation, and disposal of laboratory specimens.	1	2	3	4	5
SL5	I am familiar with the emergency procedures for laboratory fires, chemical exposures, and biohazard incidents.	1	2	3	4	5
SL6	I consistently wear appropriate personal protective equipment (PPE) during all laboratory procedures.	1	2	3	4	5
SL7	I understand the importance of timely critical value notification and can accurately report critical values to clinicians.	1	2	3	4	5
SL8	I am able to correctly classify and manage laboratory waste according to institutional safety policies.	1	2	3	4	5
SL9	I consistently follow hand hygiene protocols before and after patient contact or specimen handling.	1	2	3	4	5
SL10	I understand the principles of infection control and their application in the clinical laboratory setting.	1	2	3	4	5
SL11	I am knowledgeable about the reporting system for adverse events and near-misses in the laboratory.	1	2	3	4	5
SL12	I consistently ensure proper labeling and identification of all specimen containers.	1	2	3	4	5

Dimension 3: Patient-Centered Service Literacy (12 items)

No.	Item	1	2	3	4	5
PSL1	I am able to communicate effectively with patients during phlebotomy or specimen collection procedures.	1	2	3	4	5
PSL2	I consistently explain specimen collection procedures to patients in a clear and reassuring manner.	1	2	3	4	5
PSL3	I demonstrate empathy and respect when interacting with patients from diverse cultural backgrounds.	1	2	3	4	5
PSL4	I am able to address patients' questions and concerns regarding their laboratory tests and results.	1	2	3	4	5
PSL5	I maintain patient confidentiality and privacy in all clinical and laboratory settings.	1	2	3	4	5
PSL6	I actively seek to understand patients' perspectives and needs when providing laboratory services.	1	2	3	4	5
PSL7	I am able to manage patient anxiety and discomfort during venipuncture or other invasive procedures.	1	2	3	4	5
PSL8	I consistently provide clear post-collection instructions to patients (e.g., wound care, medication effects).	1	2	3	4	5
PSL9	I collaborate effectively with other healthcare professionals to ensure optimal patient outcomes.	1	2	3	4	5
PSL10	I understand the clinical significance of laboratory results and can explain their implications to patients in plain language.	1	2	3	4	5
PSL11	I actively seek feedback from patients to improve the quality of laboratory services I provide.	1	2	3	4	5
PSL12	I am committed to delivering patient-centered care that respects patients' values and preferences.	1	2	3	4	5

Scoring Summary

Dimension	Number of Items	Possible Score Range
Quality Literacy	12	12 – 60
Safety Literacy	12	12 – 60
Patient-Centered Service Literacy	12	12 – 60
Total Scale	36	36 – 180

Administration Notes:

- The scale is self-administered and takes approximately 5–10 minutes to complete.
- Items are scored using a 5-point Likert scale (1 = Strongly Disagree to 5 = Strongly Agree), with no reverse-scored items.
- Total scores are calculated by summing all item responses.

Supplementary Material S8:

Psychometric Properties of the Triple Helix Integrated Literacy Scale

Psychometric Property	Assessment Method	Result	Acceptable Threshold	Interpretation
Content Validity	Item-level CVI (I-CVI)	0.80–1.00	≥ 0.78	Excellent
	Scale-level CVI (S-CVI/Ave)	0.91	≥ 0.90	Excellent
Item Analysis	Item-total correlation	≥ 0.30 for all retained items	≥ 0.30	Acceptable
Construct Validity	EFA factor loadings	0.52–0.81	≥ 0.40	Good
	Eigenvalues (3 factors)	> 1.0	> 1.0	Supported
	Variance explained	62.4%	≥ 50%	Good
Internal Consistency	Cronbach's α (total scale)	0.87	≥ 0.70	Good
Test-Retest Reliability	ICC (2-week interval)	0.84 (95% CI: 0.72–0.92)	≥ 0.70	Good
Criterion-related Validity	Correlation with Mini-CEX comprehensive score	r = 0.42 (P < 0.01)	≥ 0.30 (exploratory)	Preliminary evidence

Abbreviations: CVI, content validity index; EFA, exploratory factor analysis; ICC, intraclass correlation coefficient; CI, confidence interval.

Supplementary Material S9: Mini-CEX Rating Form with Behavioral Anchors

Mini-CEX Domains and 9-Point Behavioral Anchors

**Domain**	**1–3 (Unsatisfactory)**	**4–6 (Satisfactory)**	**7–9 (Superior)**
Professionalism	Fails to demonstrate professional behavior (e.g., tardiness, inappropriate dress, disrespectful communication).	Generally professional with occasional minor lapses.	Consistently exemplary professional conduct; serves as a role model.
Communication Skills	Unclear or ineffective communication with patients or colleagues; information is poorly tailored.	Clear communication with minor deficiencies; generally effective.	Excellent communication skills; actively listens and tailors information appropriately to the audience.
Quality Awareness	Fails to recognize quality issues in laboratory processes; does not respond to quality deviations.	Identifies quality issues but does not initiate corrective actions.	Proactively identifies, reports, and suggests corrective actions for quality issues.
Safety Behaviors	Fails to adhere to safety protocols (e.g., hand hygiene, PPE use, specimen handling).	Generally adheres to safety protocols with occasional reminders needed.	Consistently adheres to and promotes safety protocols; encourages others to follow.
Patient Service	Patient interactions are abrupt, dismissive, or lack empathy.	Courteous but does not actively address patient concerns.	Demonstrates empathy; actively addresses patient concerns and ensures patient comfort during procedures.

Scoring: 1–3 = Unsatisfactory; 4–6 = Satisfactory; 7–9 = Superior. Composite score = mean of five domains. One assessment per rotation (six total); overall score = mean across all encounters.
